# Epidemiology of primary sclerosing cholangitis in general and IBD populations: a systematic review and meta-analysis

**DOI:** 10.1007/s12072-026-11127-y

**Published:** 2026-07-11

**Authors:** Glenn Jun Kit Ho, Asvin Selvakumar, Lucas Yang Yuxu Yeo, Mako Eguchi, Ryan Yanzhe Lim, Benedix Kuan Loon Sim, Daniel Tung, Apoorva N Sasikumar, Shirley Huey Shin Bong, Ethan Kai Jun Tham, Jia Hao Law, Eunice Xiang-Xuan Tan, Brian J. Wentworth, Won-Mook Choi, Aparna Goel, David N. Assis, Benjamin Nah, Vincent L. Chen

**Affiliations:** 1 Yong Loo Lin School of Medicine, National University of Singapore, Singapore, Singapore; 2 National University Centre for Digestive Health, National University Hospital, Singapore, Singapore; 3 Faculty of Medicine, Division of Metabolism and Endocrinology, Saga University, Saga, Japan; 4 Department of Medicine, Division of Gastroenterology, Ng Teng Fong General Hospital, Singapore, Singapore; 5 Department of Surgery, Division of Hepatobiliary and Pancreatic Surgery, National University Hospital, Singapore, Singapore; 6 Department of Surgery, Division of Hepatobiliary and Pancreatic Surgery, Alexandra Hospital, Singapore, Singapore; 7 National University Centre for Organ Transplantation, National University Hospital, Singapore, Singapore; 8 Division of Gastroenterology and Hepatology, University of Virginia School of Medicine, Charlottesville, VA USA; 9 Department of Gastroenterology, Liver Center, Asan Medical Center, University of Ulsan College of Medicine, Seoul, South Korea; 10 Division of Gastroenterology and Hepatology, Stanford University, Stanford, CA USA; 11 Section of Digestive Diseases, Yale School of Medicine, New Haven, CT USA; 12 Department of Medicine, Division of Gastroenterology and Hepatology, Alexandra Hospital, Tower Block Level 10, 1E Kent Ridge Road, Singapore, 119228 Singapore; 13 Department of Internal Medicine, Division of Gastroenterology and Hepatology, University of Michigan Medical School, 1500 East Medical Center Drive, Ann Arbor, MI 48109-2435 USA

**Keywords:** Primary sclerosing cholangitis, Inflammatory bowel disease, Ulcerative colitis, Crohn’s disease, Prevalence, Incidence

## Abstract

**Background & aims:**

Primary Sclerosing Cholangitis (PSC) is a chronic cholestatic liver condition that is closely associated with inflammatory bowel disease (IBD). PSC is associated with substantial long-term hepatobiliary morbidity, including an increased risk of cholangiocarcinoma. Reported prevalence and incidence estimates of PSC vary widely across different populations and study designs. This meta-analysis aims to provide an updated estimate of the prevalence of PSC in the general and IBD populations, and PSC incidence in the general population.

**Methods:**

A systematic search of MEDLINE (Ovid) and Embase from inception to October 20, 2025, for population- or cohort-based studies reporting PSC prevalence or incidence was performed. Seventy-four studies met the inclusion criteria. Random-effects meta-analyses were performed to pool prevalence and incidence estimates. Subgroup analyses, meta-regression, and sensitivity analyses were conducted to assess robustness and sources of heterogeneity.

**Results:**

Seventeen studies including 196,635,709 individuals showed a pooled overall PSC prevalence of 8.06 per 100,000 (95% CI 4.75–13.36) in the general population. Among 516,548 patients with IBD in thirty-four studies, pooled PSC prevalence was 15.2 per 1,000 (95% CI 10.7–21.7). PSC prevalence was higher in ulcerative colitis (20.6 per 1,000) than in Crohn’s disease (10.2 per 1,000). Using Crohn’s disease as the reference group, ulcerative colitis was associated with higher odds of PSC (OR 1.787, 95% CI 1.066–2.996, *p* = 0.028). Thirteen studies reported a pooled incidence of PSC in the general population of 0.892 per 100,000 person-years (95% CI 0.705–1.130).

**Conclusion:**

This meta-analysis provides an updated synthesis of PSC epidemiology, extending prior work by incorporating detailed subgroup analyses across IBD phenotype, PSC subtype, geographic region, socioeconomic context, diagnostic modality, case-ascertainment method, and time period, which are dimensions not comprehensively addressed in previous meta-analyses. PSC remains a rare disease in the general population but is substantially more prevalent among individuals with IBD. Marked geographic and methodological heterogeneity, with notable underrepresentation of data from lower-SDI regions and the African Region, underscores the need for standardized case definitions and improved epidemiological surveillance across diverse settings. Given the markedly increased risk of cholangiocarcinoma in PSC, these findings highlight the clinical importance of recognizing PSC in higher-risk IBD populations and provide an epidemiological foundation for future studies evaluating risk stratification, early detection, and surveillance.

**Supplementary Information:**

The online version contains supplementary material available at https://doi.org/10.1007/s12072-026-11127-y.

## Introduction

Primary sclerosing cholangitis (PSC) is a rare and progressive cholestatic liver disease characterized by inflammation and fibrosis affecting the intrahepatic and/or extrahepatic bile ducts. Over time, PSC can lead to the formation of multifocal biliary strictures and progressive liver dysfunction, resulting in liver cirrhosis and substantially increasing the risk of cholangiocarcinoma [1, 2]. Moreover, PSC is strongly associated with inflammatory bowel disease (IBD), hypothesized to involve a complex interplay of genetic susceptibility, gut microbiota dysbiosis, and altered bile acid homeostasis [3]. Current literature estimates that 70% of patients with PSC have concomitant IBD, especially ulcerative colitis (UC) [4]. The PSC–IBD phenotype presents a distinct clinical phenotype, frequently with endoscopic findings of right-sided colitis and backwash ileitis and is linked to increased risks of colorectal cancer, necessitating annual endoscopic surveillance [5, 6]. To date, no effective pharmacological therapies are approved for PSC to improve transplant-free survival. Liver transplantation remains the only curative option for patients with PSC-related decompensated cirrhosis, unresectable cholangiocarcinoma, and in selected patients with refractory recurrent acute cholangitis [7].

Accurate epidemiological data are imperative to benchmark disease burden, system-level healthcare utilization, and to identify at-risk patients to guide cost-effective healthcare resource allocation. However, the prevalence of PSC in the general population varies widely in the current literature, ranging from 0 to 31.7 per 100,000 [8]. In addition to geographic and ethnic differences, heterogeneity in study design and population as well as varied diagnostic and case-ascertainment methods contribute to the wide pooled estimates, thereby limiting the accuracy of epidemiological benchmarking and risk stratification for clinical and health policy decision-making. Few studies have explored the impact of time-period and case-ascertainment methods on epidemiological estimates. Furthermore, the extent to which socioeconomic context contributes to between-study differences remains poorly characterized.

Therefore, we conducted a systematic review and meta-analysis to examine the epidemiological patterns of PSC across included populations with the aims of (i) deriving overall pooled prevalence estimates for the general population and patients with IBD and pooled incidence estimates in the general population, (ii) investigating the impact of methodological variations between studies on epidemiological estimates, and (iii) describing geographical, socioeconomic, and temporal relations.

## Methods

### Search strategy

This meta-analysis was conducted in accordance with the Preferred Reporting Items for Systematic Reviews and Meta-Analyses (PRISMA) statement [9]. A comprehensive search was performed on MEDLINE (Ovid) and Embase with relevant papers included from inception to October 20, 2025. Briefly, search terms included but were not limited to *“primary sclerosing cholangitis,” “prevalence,” “incidence,” “epidemiology,” and “surveillance.”* The search strategy is provided in Supplementary Material 1. The references were imported to EndNote 21 for removal of duplicates, and bibliographies of relevant articles and previous meta-analyses were manually screened to identify additional eligible studies.

### Eligibility and selection criteria

To assess eligibility of inclusion, six authors (GH, AS, LY, RL, BS, DT) conducted independent screening of titles, abstracts, and subsequently full-text assessment. Discrepancies between authors were solved by consensus or in discussion with a senior author (VLC). The inclusion criteria included original studies with population- or cohort-level prevalence or incidence of PSC. Articles written in or fully translated into English were included in this meta-analysis. Studies reporting prevalence or incidence data exclusively in pediatric populations (< 18 years old), case series without denominators, case–control studies, conference abstracts, animal studies, meta-analyses, and systematic reviews lacking primary data were excluded. Conference abstracts were excluded because they lacked sufficient methodological detail and complete outcome reporting for reliable quality assessment and standardized data extraction. Studies defining PSC based on self-reporting and studies with solely autoimmune disease cohorts or transplant cohorts were also excluded. Studies with overlapping databases were carefully screened to ensure only the most comprehensive and up-to-date results were included; preference was given to studies that had the larger patient cohorts. A summary of included studies is shown in Supplementary Table 1.

### Data extraction

Data from the included studies were independently extracted in pairs by six blinded authors (GH, AS, LY, RL, BS, DT) using a predefined, standardized data extraction form. Discrepancies were resolved by consensus. Extracted study-level variables included author, year of publication (categorized as pre-2018 vs. 2018 onward), country, World Health Organization (WHO) region, and socio-demographic index (SDI). Methodological characteristics recorded were study design, study period, sample size, number of PSC cases, and person-time at risk where applicable.

Clinical variables extracted included PSC phenotype (large-duct or small-duct), IBD subtype (ulcerative colitis or Crohn’s disease), disease location, sex distribution, diagnostic modality for PSC (imaging, biopsy, International Classification of Diseases (ICD) codes, or combined approaches), and case-ascertainment method (administrative databases vs. hospital- or clinic-based cohorts). Patients with IBD-unclassified (IBD-U), when reported, were included in the overall IBD analysis but not analyzed as a separate subtype. Imaging-based diagnosis was defined as the use of endoscopic retrograde cholangiopancreatography (ERCP) or magnetic resonance cholangiopancreatography (MRCP). The primary outcomes were the pooled prevalence of PSC in the general population and among individuals with IBD, ulcerative colitis, and Crohn’s disease. Secondary outcomes included PSC prevalence stratified by PSC phenotype, IBD subtype, disease locations, sex, WHO Region, SDI, study period, case-ascertainment method, and diagnostic modality. Due to limited available data, incidence analyses were restricted to the general population. In this review, “general population” refers to population-based samples without restriction by IBD status, rather than a confirmed non-IBD population.

### Statistical analysis

Statistical analyses were performed using R (version 4.4.1), and a two-sided p-value < 0.05 was considered statistically significant. A meta-analysis of proportions was performed to estimate the prevalence of PSC using the *metaprop* function, applying a Freeman–Tukey double arcsine transformation and a random-effects model [10, 11]. Incidence rates of PSC were pooled using a random-effects meta-analysis of incidence rates, using the *metarate* function, employing log incidence rate as the summary measure. Between-study heterogeneity was estimated using restricted maximum likelihood (REML) [12]. Statistical heterogeneity was quantified using the I^2^ statistic [13], with values of 25%, 50%, and 75% representing low, moderate, and high heterogeneity, respectively. Subgroup analyses were conducted to assess sources of heterogeneity across populations. Regional subgrouping was done by identifying the country of the study population and classifying studies into six regions (Europe, South-East Asia, Western Pacific, Africa, the Americas and the Eastern Mediterranean Region), and five socio-demographic index classes (High, High-Middle, Middle, Low-Middle, Low) based on the World Health Organization (WHO) Region and Institute for Health Metrics and Evaluation (IHME) classification, respectively [14, 15]. Additional subgroup analyses stratified PSC prevalence by IBD subtypes and disease location according to the Montreal Classification of IBD, including ulcerative colitis extent (proctitis, pancolitis, left-sided colitis) and Crohn’s disease location (ileal, colonic, and ileocolonic involvement), where data were available [16]. Given anticipated variability in case definitions, diagnostic modalities, and underlying population characteristics across settings, substantial between-study heterogeneity was expected. We also anticipated misclassification in administrative database studies because a specific ICD-10-CM code for PSC (K83.01) was only introduced in 2018. Earlier database studies may have relied on less specific cholangitis or biliary codes, potentially affecting case ascertainment. Considering this, we stratified studies by publication period (before 2018 vs. 2018 onwards) to account for changes in PSC ascertainment following the introduction of the ICD-10-CM Code. Factors associated with PSC prevalence across studies were evaluated using random-effects meta-regression models implemented with the *metafor* package in R. Study-specific prevalence estimates were logit-transformed to stabilize variances and improve normality assumptions, with study-level PSC prevalence proportions used as the dependent variable.

### Quality of included articles

The quality of included articles was assessed using the Joanna Briggs Institute (JBI) critical appraisal tool [17]. Potential sources of bias included (i) whether exposed and non-exposed cohorts were drawn from the same population, (ii) adequate assessment of exposure, (iii) confidence that the outcome was not present at baseline, (iv) appropriate matching of exposed and unexposed cohorts and/or adequate adjustment for differences, (v) confidence in prognostic factor assessment, (vi) confidence in outcome assessment, and (vii) adequate follow-up.

### Sensitivity analyses

Several sensitivity analyses were conducted to assess the robustness of pooled estimates. First, studies utilizing ICD codes as the sole diagnostic method were excluded, given concerns that administrative coding may misclassify PSC diagnoses and underestimate true prevalence. Second, pre-2018 administrative database studies were excluded as ICD-10-CM codes for PSC were not available prior to 2018, meaning administrative studies conducted before this period could not reliably identify PSC cases, potentially introducing misclassification bias into prevalence and incidence estimates. Third, a leave-one-out analysis was performed whereby each study was sequentially omitted and the remaining studies re-pooled, to identify whether any single study exerted undue influence on the pooled estimate. Fourth, a sensitivity analysis restricted to high-quality studies was conducted, defined as those achieving a JBI critical appraisal score of ≥ 80% of applicable items, to assess whether study quality influenced the robustness of pooled estimates. Items rated “Not Applicable” were excluded from the denominator when calculating the JBI score. Finally, prediction intervals were calculated for all pooled estimates to quantify the expected range of true prevalence or incidence rates in a new population, providing a measure of between-study heterogeneity. Prediction intervals were only reported for subgroups with five or more studies, as between-study variance is unreliable with fewer studies; where substantial heterogeneity is present, prediction intervals should be interpreted with caution given the potential for wide and imprecise bounds. Prevalence in the general population is reported per 100,000 persons, while prevalence in IBD, ulcerative colitis, and Crohn’s disease populations is reported per 1,000 persons.

## Results

### Search results

**Fig. 1:**
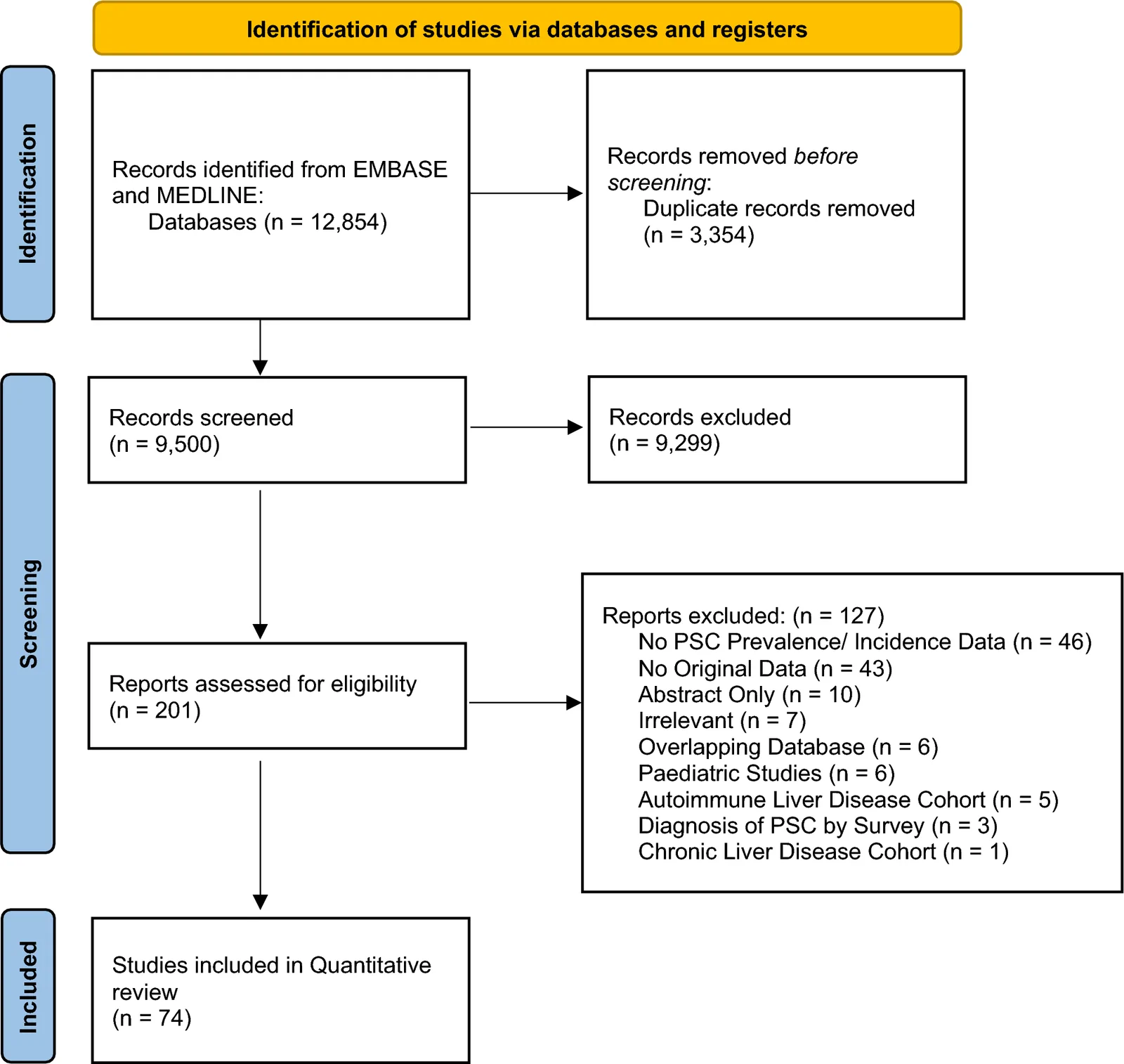
PRISMA flowchart of included articles

A total of 5,918 records from MEDLINE (Ovid) and 6,936 records from Embase were retrieved from the initial search. 9,500 records were screened after 3,354 duplicates were removed. 9,299 articles were excluded based on study title and abstract and the remaining 201 articles were retrieved for full-text review, of which a final 74 studies met inclusion criteria. The flowchart of studies included is depicted in Fig. 1. Overall, the included studies were moderate-to-high quality on the Joanna Briggs Institute (JBI) critical appraisal tools, with most items rated “Yes” across cohort, cross-sectional, prevalence, case–control, and case-series designs. 62 out of 74 of the included studies had a JBI score of ≥ 80%. The results of the quality assessment for all included studies are presented in Supplementary Table 2.

### Prevalence of PSC in general population

There were a total of seventeen studies describing the prevalence of PSC in the general population, comprising 196,635,709 individuals, with a total of 6,091 cases of PSC. The pooled prevalence of PSC in the general population was 8.06 per 100,000 (95% CI 4.75–13.36, I^2^ = 100%) (Table 1). The prediction interval for the overall prevalence was wide (0.57–57.34 per 100,000), reflecting substantial between-study heterogeneity. Large-duct PSC demonstrated higher prevalence than small-duct PSC with pooled prevalence of 11.10 per 100,000 (95% CI 4.16–26.45, I^2^ = 100%) vs. 1.24 per 100,000 (95% CI 0.45–3.35, I^2^ = 100%) (*p* = 0.002) (Table 1, Supplementary Material 2). Prevalence data also varied depending on diagnostic method, where pooled prevalence estimates were higher in studies using imaging (15.19 per 100,000, 95% CI 8.98–24.54, I^2^ = 100%), compared to studies using both imaging and biopsy (6.90 per 100,000, 95% CI 3.32–13.79, I^2^ = 100%) (*p* = 0.001) (Table 1). Studies from 2018 onwards showed a higher pooled prevalence of PSC (9.35 per 100,000, 95% CI 5.12–16.49, I^2^ = 100%) compared to studies before 2018 (7.05 per 100,000, 95% CI 2.98–15.78, I^2^ = 100%), although these differences were not statistically significant (*p* = 0.588). Comparison of prevalence estimates among the different WHO Regions, SDIs, case-ascertainment methods also showed no statistically significant differences.

**Table 1:** Prevalence of primary sclerosing cholangitis in the general population

	No. of studies	Sample Size	Cases	Pooled prevalence per 100,000 [95% confidence interval]	I^2^	Prediction interval^††^	Subgroup difference
Overall	17	196,635,709	6,091	8.06 [4.75 – 13.36]	100.0%	0.57 – 57.34	
Sensitivity analyses							
Excluding pre-2018 administrative studies	15	179,723,144	5,114	8.55 [4.59 – 15.37]	100.0%	0.50 – 63.67	
High-quality studies (JBI ≥ 80%)	16	196,572,073	6,084	7.90 [4.43—13.71]	100.0%	0.50 – 59.35	
Phenotype							
Small-Duct PSC	2	10,066,764	64	1.24 [0.45—3.35]	100.0%	−^†^	*p* = 0.002*
Large-Duct PSC	2	10,066,764	582	11.10 [4.16—26.45]	100.0%	−^†^	
WHO Region							
European Region	10	60,555,319	3,182	6.98 [3.32—14.08]	100.0%	0.30 – 64.85	p = 0.314
Region of the Americas	3	3,477,845	199	16.69 [5.66 – 40.11]	100.0%	−^†^	
Western Pacific Region	4	132,602,545	2,710	6.50 [3.20 – 12.75]	100.0%	−^†^	
Socio-Demographic Index							
High	15	177,397,389	6,045	9.00 [6.26 – 12.77]	100.0%	1.67 – 36.51	*p* = 0.450
High-Middle	2	19,238,320	46	3.43 [0.26 – 32.94]	100.0%	−^†^	
Dates							
Before 2018	9	48,157,783	1,919	7.05 [2.98—15.78]	100.0%	0.22 – 72.60	*p* = 0.588
2018 Onwards	8	148,477,926	4,172	9.35 [5.12—16.49]	100.0%	0.82 – 56.43	
Case-Ascertainment Method							
Administrative database studies	5	31,054,994	1,809	6.38 [4.53 – 8.90]	100.0%	1.60 – 22.20	*p* = 0.376
Hospital/ Clinic-based Studies	12	165,580,715	4,282	8.87 [4.60 – 16.43]	100.0%	0.35 – 73.03	
Diagnosis Method							
Imaging	4	3,485,722	855	15.19 [8.98 – 24.54]	100.0%	−^†^	*p* = 0.001*
Imaging and Biopsy	11	170,883,095	4,046	6.90 [3.32 – 13.79]	100.0%	0.27 – 67.29	
Others/ Unspecified	2	22,266,892	1,190	5.02 [3.80 – 6.60]	100.0%	−^†^	

*WHO* World Health Organization; *JBI* Joanna Briggs Institute

^*^: *p* < 0.05 denotes statistical significance

^†^: Prediction intervals not reported for subgroups with *k* < 5 studies due to unreliable estimation of between-study variance

^††^: Prediction intervals should be interpreted with caution due to substantial between-study heterogeneity, which may result in imprecise estimation of between-study variance and wide prediction bounds

### Prevalence of PSC in IBD population

**Fig. 2:**
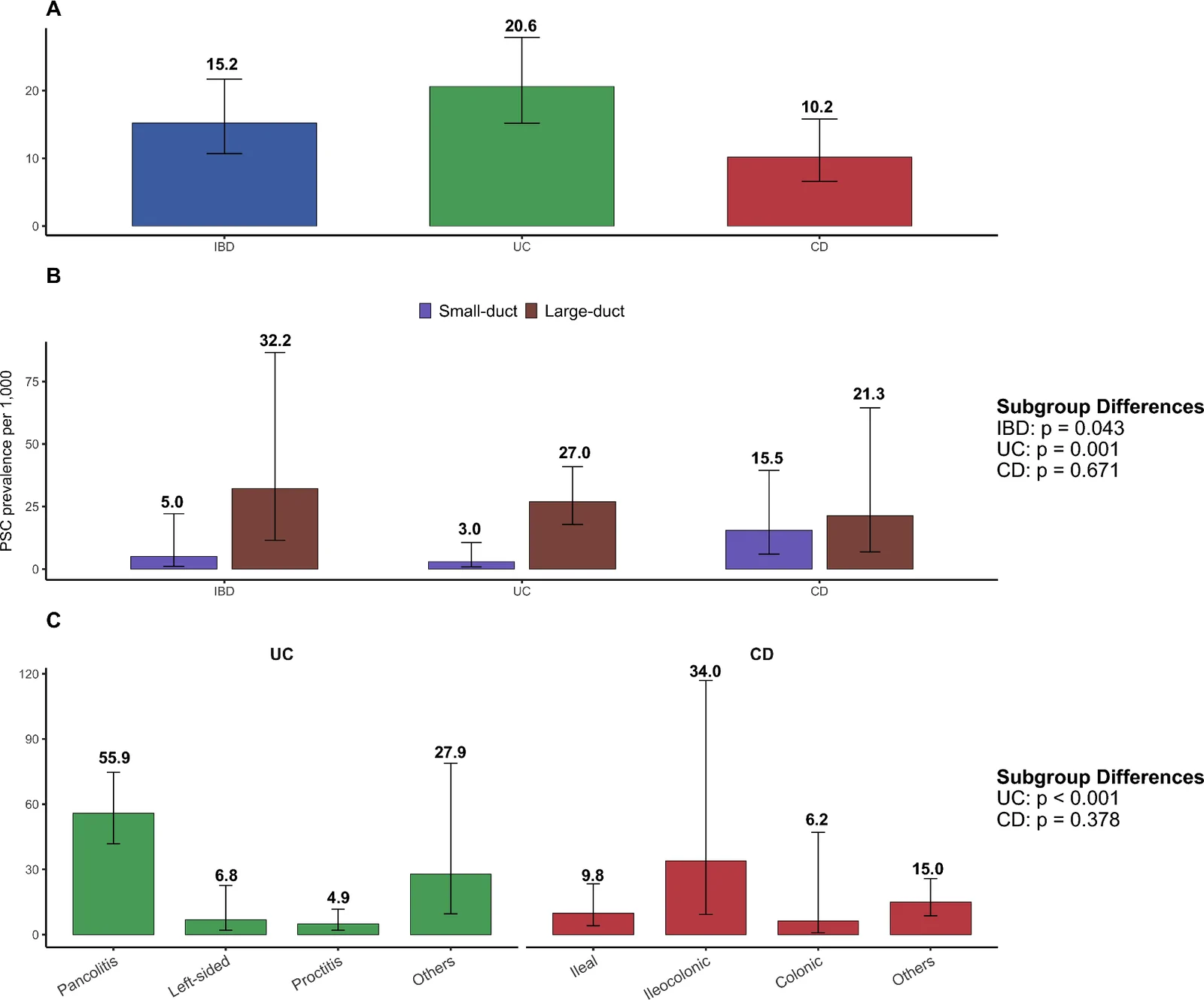
Prevalence of Primary Sclerosing Cholangitis (PSC) by disease group and subtype in inflammatory bowel disease (IBD) (per 1,000): **A** overall IBD, ulcerative colitis (UC), and Crohn’s disease (CD); **B** small-duct and large-duct PSC; **C** disease distribution in UC and CD

**Fig. 3:**
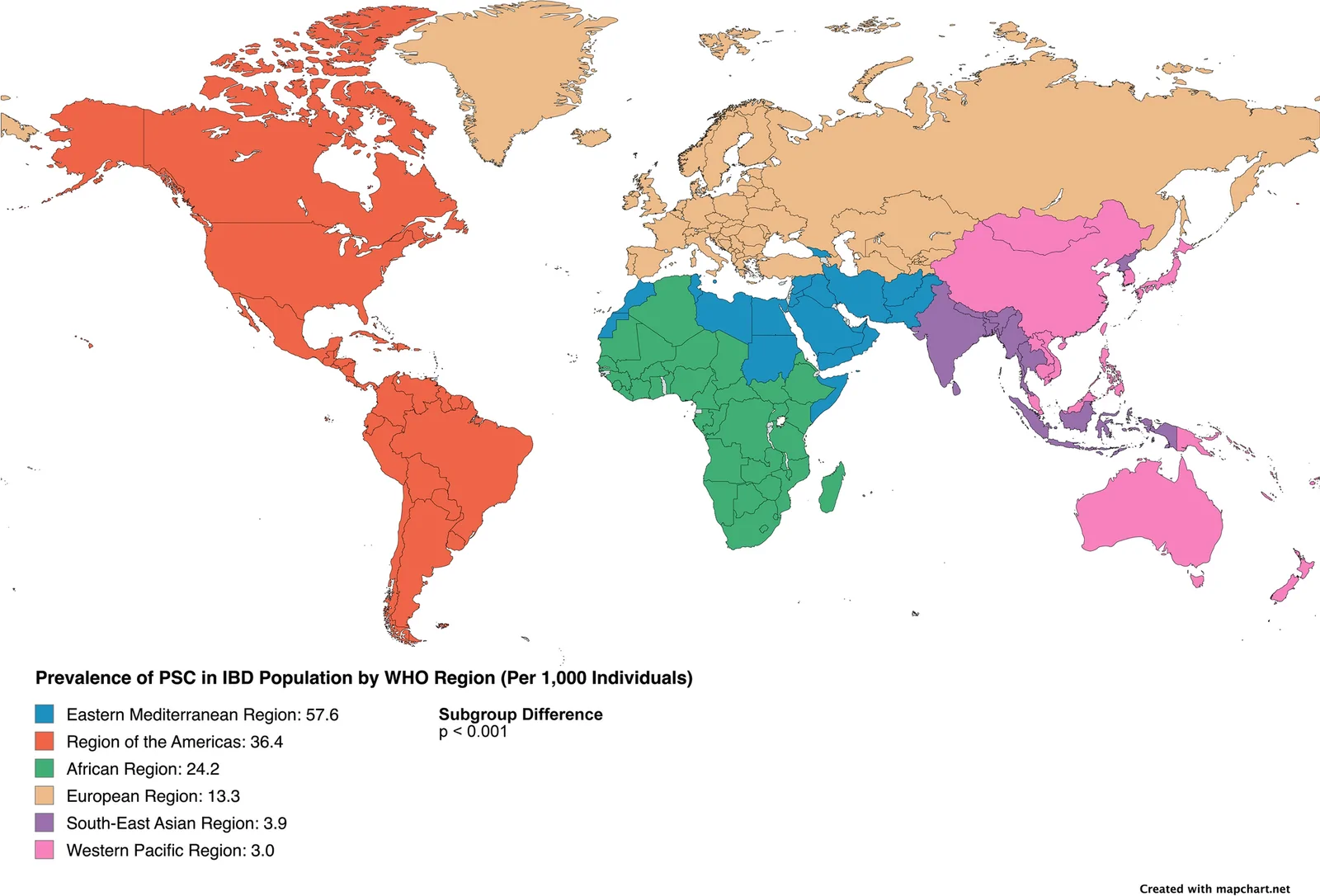
Regional prevalence of primary sclerosing cholangitis among patients with inflammatory bowel disease by WHO Region

**Fig. 4:**
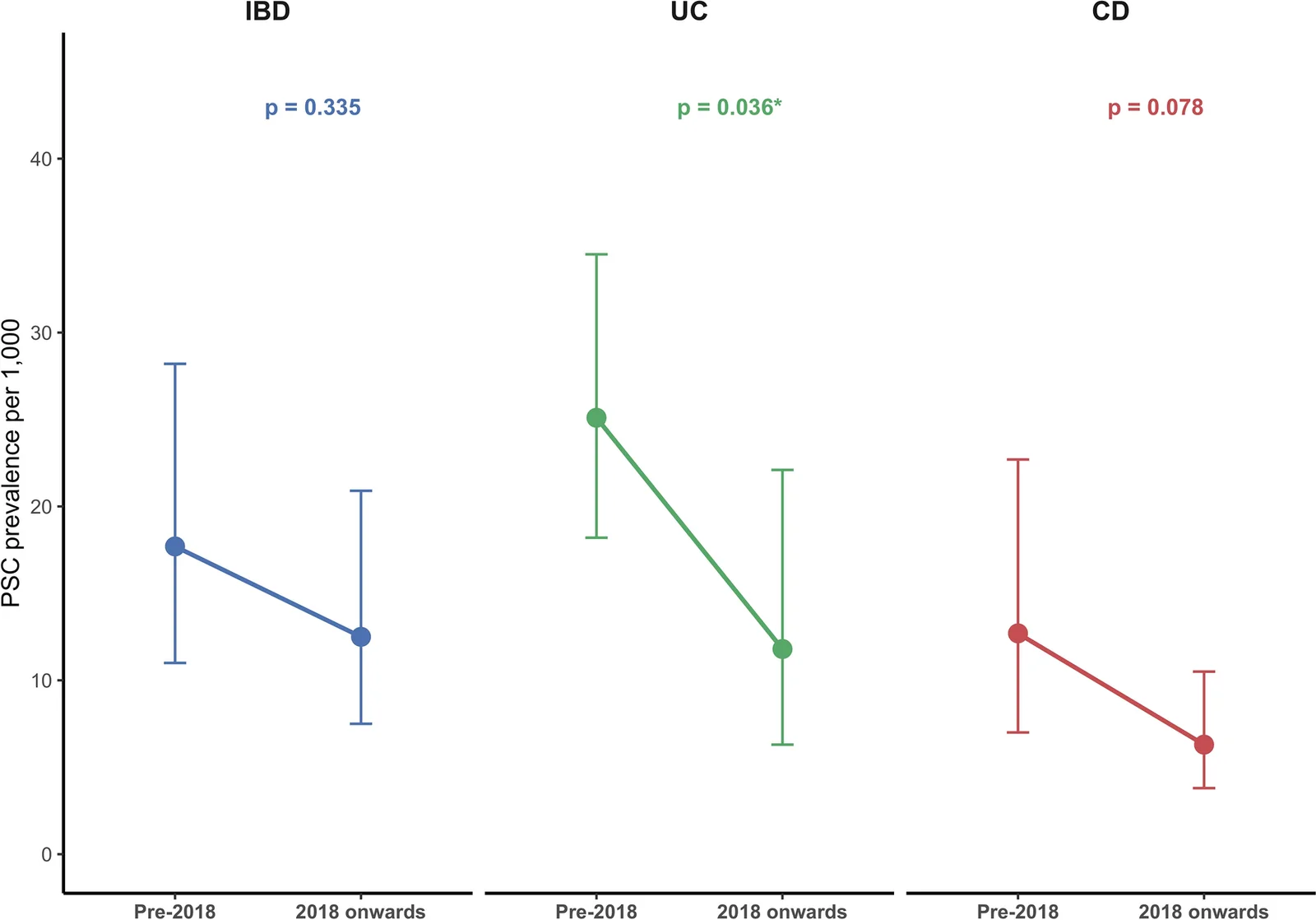
Temporal trends in primary sclerosing cholangitis prevalence across inflammatory bowel disease subtypes

Thirty-four studies comprising 516,548 IBD patients with a total of 4,970 PSC cases yielded a pooled prevalence of 15.2 per 1,000 (95% CI 10.7–21.7, I^2^ = 98.0%). The prediction interval was 1.9–113.4 per 1,000, reflecting the wide variability in PSC prevalence expected across different IBD populations. The prevalence of large-duct PSC was over six times more common in the IBD population (32.2 per 1,000, 95% CI 11.5–86.5, I^2^ = 94.4%) compared to small-duct PSC (5.0 per 1,000, 95% CI 1.1–22.1, I^2^ = 5.9%) (*p* = 0.043) (Table 2, Fig. 2). Sex-stratified analysis showed a greater pooled prevalence of 23.7 per 1,000 (95% CI 9.3–58.9, I^2^ = 98.2%) among males compared to 15.8 per 1,000 (95% CI 6.8–36.3, I^2^ = 96.4%) among females (Table 2). When stratified by WHO Region, PSC prevalence was highest in the Eastern Mediterranean Region with pooled prevalence of 57.6 per 1,000 (95% CI 45.2–73.0, I^2^ = 33.2%), followed by the Region of the Americas (36.4 per 1,000, 95% CI 21.0–62.4, I^2^ = 96.7%), and lowest in the Western Pacific Region with prevalence of 3.0 per 1,000 (95% CI 2.5–3.5, I^2^ = 0.0%) (*p* < 0.001) (Table 2, Fig. 3). According to Socio-demographic index (SDI), PSC had the highest prevalence in High-Middle SDI countries (21.6 per 1,000, 95% CI 12.5–37.1, I^2^ = 97.1%) and lowest prevalence in Low-Middle SDI countries (3.9 per 1,000, 95% CI 3.0–5.1, I^2^ = 0.0%) (*p* < 0.001) (Table 2). Studies using imaging-based diagnosis displayed the highest pooled prevalence of 21.9 per 1,000 (95% CI 11.0–43.2, I^2^ = 96.9%), followed by studies using both imaging and biopsy (18.6 per 1,000, 95% CI 10.2–33.7, I^2^ = 98.7%), studies with unspecified or other diagnostic methods (12.4 per 1,000, 95% CI 7.4–20.6, I^2^ = 90.1%), and studies using ICD code (5.3 per 1,000, 95% CI 3.1–8.8, I^2^ = 97.4%) (*p* = 0.002) (Table 2). Hospital or clinic-based studies showed higher PSC prevalence (17.4 per 1,000, 95% CI 11.4–26.4, I^2^ = 95.9%) compared to administrative database studies (10.8 per 1,000, 95% CI 6.0–19.5, I^2^ = 99.1%), although this difference was not statistically significant (*p* = 0.199) (Table 2). Studies before 2018 reported higher pooled PSC prevalence (17.7 per 1,000, 95% CI 11.0–28.2, I^2^ = 97.1%) than studies from 2018 onwards (12.5 per 1,000, 95% CI 7.5–20.9, I^2^ = 98.7%), although these findings were again not statistically significant (*p* = 0.335) (Table 2, Fig. 4).

**Table 2:** Prevalence of primary sclerosing cholangitis in inflammatory bowel disease population

	No. of studies	Sample size	Cases	Pooled prevalence per 1,000 [95% confidence interval]	I^2^	Prediction interval^††^	Subgroup difference
Overall	34	516,548	4,970	15.2 [10.7 – 21.7]	98.0% [97.7—98.3]	1.9 – 113.4	
Sensitivity analyses							
Excluding pre-2018 administrative studies	30	465,012	4,621	15.1 [10.4 – 22.0]	98.0% [97.6 – 98.3]	1.8 – 113.0	
Excluding ICD-only studies	31	413,317	4,468	17.1 [11.9 – 24.5]	97.7% [97.2 – 98.0]	2.2 – 120.1	
High-quality studies (JBI ≥ 80%)	28	513,575	4,889	14.1 [9.5–20.9]	98.3% [98.0–98.5]	1.6–113.1	
Phenotype							
Small-Duct PSC	4	2,034	20	5.0 [1.1 – 22.1]	5.9%	−^†^	*p* = 0.043*
Large-Duct PSC	4	2,034	65	32.2 [11.5 – 86.5]	94.4%	−^†^	
Gender							
Male	8	30,416	181	23.7 [9.3 – 58.9]	98.2% [97.5 – 98.7]	0.8 – 437.2	*p* < 0.001*
Female	8	19,980	117	15.8 [6.8 – 36.3]	96.4% [94.6 – 97.6]	0.8 – 249.5	
WHO region^#^							
Eastern Mediterranean Region	3	1,231	71	57.6 [45.2 – 73.0]	33.2%	−^†^	*p* < 0.001*
European Region	20	398,676	3,547	13.3 [9.0 – 19.6]	96.3%	2.1 – 78.8	
Region of the Americas	5	57,469	1,149	36.4 [21.0 – 62.4]	96.7%	4.3 – 248.8	
South-East Asia Region	2	13,665	53	3.9 [3.0 – 5.1]	0.0%	−^†^	
Western Pacific Region	2	43,329	129	3.0 [2.5 – 3.5]	0.0%	−^†^	
African Region	1	414	10	24.2 [13.0 – 44.3]	−**	−^†^	
Socio-demographic index							
High	19	450,462	4,381	15.1 [9.2 – 24.6]	98.6%	1.5 – 135.1	*p* < 0.001*
High-middle	9	49,436	488	21.6 [12.5 – 37.1]	97.1%	2.8 – 147.4	
Middle	4	2,985	48	15.3 [6.5 – 35.5]	87.6%	−^†^	
Low-middle	2	13,665	53	3.9 [3.0 – 5.1]	0.0%	−^†^	
Dates							
Before 2018	20	64,248	617	17.7 [11.0–28.2]	97.1%	2.0 – 140.0	*p* = 0.335
2018 Onwards	14	452,300	4,353	12.5 [7.5–20.9]	98.7%	1.4 – 102.4	
Case-ascertainment method							
Administrative database studies	9	468,147	4,366	10.8 [6.0 – 19.5]	99.1%	1.2 – 94.0	*p* = 0.199
Hospital/ Clinic-based Studies	25	48,401	604	17.4 [11.4 – 26.4]	95.9%	2.0 – 134.0	
Diagnosis method							
Imaging	9	18,211	221	21.9 [11.0 – 43.2]	96.9%	1.7 – 226.3	*p* = 0.002*
Imaging and Biopsy	12	379,870	4,064	18.6 [10.2 – 33.7]	98.7%	1.7 – 178.3	
ICD Code	3	103,231	502	5.3 [3.1 – 8.8]	97.4%	−^†^	
Others/ Unspecified	10	15,236	183	12.4 [7.4 – 20.6]	90.1%	2.0 – 72.1	

^*^: *p* < 0.05 denotes statistical significance

**: I^2^ not calculated for single-study subgroups

^†^: Prediction intervals not reported for subgroups with *k* < 5 studies due to unreliable estimation of between-study variance

^††^: Prediction intervals should be interpreted with caution due to substantial between-study heterogeneity, which may result in imprecise estimation of between-study variance and wide prediction bounds.

^**#**^ One study excluded for recruiting patients from centers across both Western Pacific and South-east Asian WHO regions

### Prevalence of PSC in ulcerative colitis population

A total of forty-seven studies involving 101,276 ulcerative colitis patients and 1,096 PSC cases were included in this analysis, with an overall pooled prevalence of 20.6 per 1,000 (95% CI 15.2–27.8, I^2^ = 96.6%) in the ulcerative colitis population (Supplementary Table 3, Fig. 2). With respect to ulcerative colitis disease locations, PSC prevalence was highest in pancolitis (55.9 per 1,000, 95% CI 41.7–74.7, I^2^ = 70.5%), and lowest in proctitis (4.9 per 1,000, 95% CI 2.0–11.7, I^2^ = 0.0%) (Supplementary Table 3, Fig. 2). Large-duct PSC was more prevalent in ulcerative colitis (27.0 per 1,000, 95% CI 17.8–40.9, I^2^ = 75.9%) compared to small-duct PSC (3.0 per 1,000, 95% CI 0.9–10.6, I^2^ = 85.1%) (*p* = 0.001) (Supplementary Table 3, Fig. 2). Sex-stratified analysis demonstrated a higher pooled prevalence of PSC in males (22.5 per 1,000, 95% CI 12.1–41.6, I^2^ = 96.8%) compared to females (14.0 per 1,000, 95% CI 8.3–23.5, I^2^ = 90.2%) (Supplementary Table 3). Regionally, pooled PSC prevalence was highest in the Eastern Mediterranean Region (66.1 per 1,000, 95% CI 36.9–115.4, I^2^ = 78.1%), followed by the Region of the Americas (38.3 per 1,000, 95% CI 27.2–53.6, I^2^ = 71.8%), and lowest in the South-East Asian Region (4.5 per 1,000, 95% CI 3.4–6.0, I^2^ = 0.0%) (Supplementary Table 3). When stratified by Socio-demographic index, high-middle SDI countries reported the highest pooled PSC prevalence (29.1 per 1,000, 95% CI 17.0–49.4, I^2^ = 94.6%), followed by high-SDI countries (21.3 per 1,000, 95% CI 14.4–31.5, I^2^ = 97.4%), middle SDI countries (20.9 per 1,000, 95% CI 9.6–44.9, I^2^ = 83.8%), and low-middle SDI countries reporting the lowest PSC prevalence of 4.5 per 1,000 (95% CI 3.4–6.0, I^2^ = 0.0%) (*p* < 0.001) (Supplementary Table 3). Studies before 2018 reported a significantly higher PSC prevalence of 25.1 per 1,000 (95% CI 18.2–34.5, I^2^ = 95.7%) compared to studies from 2018 onwards (11.8 per 1,000, 95% CI 6.3–22.1, I^2^ = 96.8%) (*p* = 0.036) (Supplementary Table 3, Fig. 4). Additionally, studies that diagnosed PSC via both imaging and biopsy reported the highest prevalence estimates (26.7 per 1,000, 95% CI 17.4–40.8, I^2^ = 90.3%), followed by imaging only (26.2 per 1,000, 95% CI 15.4–44.2, I^2^ = 95.2%), other and unspecified diagnosis (14.0 per 1,000, 95% CI 7.7–25.3, I^2^ = 88.7%) and lastly ICD code (6.0 per 1,000, 95% CI 3.4–10.6, I^2^ = 96.8%) (*p* < 0.001) (Supplementary Table 3). There was no statistically significant difference reported between PSC prevalence in administrative database studies (12.3 per 1,000, 95% CI 5.4–27.7, I^2^ = 93.4%) and hospital or clinic-based studies (22.8 per 1,000, 95% CI 16.7–31.2, I^2^ = 98.1%) (*p* = 0.165) (Supplementary Table 3).

### Prevalence of PSC in Crohn’s disease population

Thirty-one studies including 46,725 patients with Crohn’s disease and 252 cases of PSC reported a pooled prevalence of 10.2 per 1,000 (95% CI 6.6–15.8, I^2^ = 91.1%) (Supplementary Table 4). When stratified by Crohn’s disease subtype, ileal disease reported a pooled PSC prevalence of 9.8 per 1,000 (95% CI 4.1–23.4, I^2^ = 46.4%), while ileocolonic disease was associated with the highest pooled PSC prevalence of 34.0 per 1,000 (95% CI 9.3–116.9, I^2^ = 87.9%). In contrast, colonic disease was associated with the lowest pooled PSC prevalence of 6.2 per 1,000 (95% CI 0.8–47.1, I^2^ = 85.4%) (Supplementary Table 4, Fig. 2). However, subgroup differences across Crohn’s disease subtypes were not statistically significant (*p* = 0.378). Large-duct PSC was more prevalent in Crohn’s disease (21.3 per 1,000, 95% CI 6.8–64.5, I^2^ = 85.3%) compared to small-duct PSC (15.5 per 1,000, 95% CI 6.0–39.4, I^2^ = 0.0%) (Supplementary Table 4, Fig. 2). Regardless, no significant difference was observed between PSC phenotypes (*p* = 0.671). Sex-stratified analysis reported pooled prevalence to be higher in males (9.7 per 1,000, 95% CI 3.3–28.5, I^2^ = 93.2%), compared to females (7.4 per 1,000, 95% CI 2.6–20.7, I^2^ = 85.3%) (Supplementary Table 4). Studies where PSC was diagnosed via imaging only again had the highest reported prevalence (13.8 per 1,000, 95% CI 6.3–30.1, I^2^ = 89.1%), followed by diagnosis via both imaging and biopsy (12.5 per 1,000, 95% CI 5.2–29.6, I^2^ = 85.5%). Diagnosis via ICD code yielded the lowest pooled prevalence of 3.5 per 1,000, 95% CI 2.0–6.3, I^2^ = 93.6%, *p* = 0.008 (Supplementary Table 4). Regionally, pooled PSC prevalence was highest in the Eastern Mediterranean Region (15.9 per 1,000, 95% CI 4.6– 53.5, I^2^ = 0.0%) and in high-middle SDI countries (13.3 per 1,000, 95% CI 8.3–21.1, I^2^ = 73.8%). In contrast, pooled PSC prevalence was lowest in the South-East Asia Region (1.9 per 1,000, 95% CI 0.8–4.1, I^2^ = 0.0%, *p* < 0.001) and low-middle SDI countries (1.9 per 1,000, 95% CI 0.8–4.1, I^2^ = 0.0%, *p* < 0.001) (Supplementary Table 4). Hospital or clinic-based studies reported numerically higher but non-significant PSC prevalence estimates (11.1 per 1,000, 95% CI 7.0–17.6, I^2^ = 81.1%) compared to administrative database studies (8.4 per 1,000, 95% CI 2.9–24.2, I^2^ = 96.5%) (*p* = 0.636) (Supplementary Table 4). Additionally, studies before 2018 reported a higher PSC prevalence (12.7 per 1,000, 95% CI 7.0–22.7, I^2^ = 91.3%) compared to studies from 2018 onwards (6.3 per 1,000, 95% CI 3.8– 10.5, I^2^ = 83.1%), although these results were not statistically significant (*p* = 0.078) (Supplementary Table 4, Fig. 4).

### Incidence of PSC in general population

Thirteen studies reported a total of 2,206 incident cases over 274,365,833 person-years, yielding a pooled incidence rate of 0.892 per 100,000 person-years (95% CI 0.705–1.130, I^2^ = 97.2%) (Supplementary Table 5, Supplementary Material 3). All incident cases originated from high-SDI countries. Pooled incidence rates were significantly higher in hospital or clinic-based studies (1.057 per 100,000 person-years, 95% CI 0.824–1.356, I^2^ = 97.0%) compared to administrative database studies (0.599 per 100,000 person-years, 95% CI 0.448–0.802, I^2^ = 87.7%) (*p* = 0.004) (Supplementary Table 5, Supplementary Material 3). Pooled incidence rates were significantly higher among studies that diagnosed PSC via imaging only (1.314 per 100,000 person-years, 95% CI 1.051–1.643, I^2^ = 78.6%) compared to studies that diagnosed PSC via both imaging and biopsy (0.823 per 100,000 person-years, 95% CI 0.583–1.163, I^2^ = 92.9%) (*p* < 0.001) (Supplementary Table 5, Supplementary Material 3). Countries from the Western Pacific Region reported greatest incidence of PSC in the general population (1.113 per 100,000 person-years, 95% CI 0.659–1.880, I^2^ = 54.2%), followed by the European Region (0.889 per 100,000 person-years, 95% CI 0.662–1.190, I^2^ = 98.1%) and the Region of the Americas (0.792 per 100,000 person-years, 95% CI 0.412–1.525, I^2^ = 95.5%) (*p* = 0.683) (Supplementary Table 5, Supplementary Material 3). Studies before 2018 reported non-significantly lower incidence of PSC (0.839 per 100,000 person-years, 95% CI 0.601–1.172, I^2^ = 95.2%) compared to studies from 2018 onwards (1.008 per 100,000 person-years, 95% CI 0.740–1.372, I^2^ = 97.1%) (*p* = 0.429) (Supplementary Table 5, Supplementary Material 3). Incidence of PSC in IBD could not be estimated due to a lack of studies on this topic.

### Meta-regression of study-level factors associated with PSC prevalence

In the general population, small-duct PSC was significantly lower in prevalence compared to large-duct PSC (OR 0.109, 95% CI 0.015–0.783, *p* = 0.028) (Table 3). In the IBD population, low-middle SDI countries had significantly lower odds of PSC compared to high-middle SDI countries (OR 0.164, 95% CI 0.034–0.806, *p* = 0.026) (Table 4). Ulcerative colitis patients also had significantly higher odds of PSC compared to Crohn’s disease patients (OR 1.787, 95% CI 1.066–2.996, *p* = 0.028), as well as IBD patients from the European Region (OR 3.776, 95% CI 1.101–12.947, *p* = 0.035), Eastern Mediterranean Region (OR 16.216, 95% CI 3.632–72.394, *p* < 0.001) and Region of the Americas (OR 10.316, 95% CI 2.613–40.724, *p* < 0.001), with reference to the South-East Asian Region (Table 4). Studies using ICD code for PSC case-ascertainment also yielded significantly lower odds of PSC (OR 0.272, 95% CI 0.079–0.933, *p* = 0.038) with reference to studies using both imaging and biopsy for PSC diagnosis (Table 4).

**Table 3:** Meta-regression evaluating factors associated with prevalence of primary sclerosing cholangitis in the general population

Variable	Odds Ratio	95% Confidence Interval	P-Value
WHO Region [Reference: Region of the Americas]			
European Region	0.454	0.091 – 2.269	*p* = 0.336
Western Pacific Region	0.433	0.068 – 2.760	*p* = 0.376
Socio-demographic index [Reference: High-Middle]			
High	4.117	0.759 – 22.332	*p* = 0.101
Case-Ascertainment Method [Reference: Administrative database studies]			
Hospital/ Clinic-based Studies	1.293	0.364 – 4.587	*p* = 0.691
Diagnosis method [Reference: Imaging and Biopsy]			
Imaging	2.368	0.607 – 9.246	*p* = 0.215
Others/ Unspecified	0.792	0.135 – 4.643	*p* = 0.796
Phenotype [Reference: Large-Duct PSC]			
Small-Duct PSC	0.109	0.015 – 0.783	*p* = 0.028*
Dates [Reference: 2018 Onwards]			
Before 2018	0.723	0.229 – 2.281	*p* = 0.580

*WHO* World Health Organization

^*^: *p* < 0.05 denotes statistical significance

**Table 4:** Meta-regression evaluating factors associated with prevalence of primary sclerosing cholangitis in inflammatory bowel disease patients

Variable	Odds ratio	95% Confidence interval	P-Value
WHO Region [Reference: South-East Asia Region]			
European Region	3.776	1.101 – 12.947	*p* = 0.035*
Eastern Mediterranean Region	16.216	3.632 – 72.394	*p* < 0.001*
Region of the Americas	10.316	2.613 – 40.724	*p* < 0.001*
African Region	6.644	0.865 – 51.050	*p* = 0.069
Western Pacific Region	1.018	0.164 – 6.314	*p* = 0.985
Socio-Demographic Index [Reference: High-Middle]			
High	0.708	0.315 – 1.594	*p* = 0.404
Middle	0.754	0.208 – 2.732	*p* = 0.668
Low-Middle	0.164	0.034 – 0.806	*p* = 0.026*
Case-Ascertainment Method [Reference: Administrative database studies]			
Hospital/ Clinic-based Studies	1.708	0.780 – 3.741	*p* = 0.180
IBD Subtype [Reference: Crohn’s Disease]			
Ulcerative Colitis	1.787	1.066 – 2.996	*p* = 0.028*
Diagnosis Method [Reference: Imaging and Biopsy]			
Imaging	1.214	0.506 – 2.911	*p* = 0.665
ICD Code	0.272	0.079 – 0.933	*p* = 0.038*
Others/ Unspecified	0.673	0.285 – 1.590	*p* = 0.367
Phenotype [Reference: Large-Duct PSC]			
Small-Duct PSC	0.216	0.038 – 1.221	*p* = 0.083
Dates [Reference: 2018 Onwards]			
Before 2018	1.497	0.730 – 3.070	*p* = 0.271

*WHO* World Health Organization

^*^: *p* < 0.05 denotes statistical significance

Among patients with ulcerative colitis, compared to South-East Asian Region, studies from the European Region (OR 5.335, 95% CI 1.637–17.385, *p* = 0.005), Eastern Mediterranean Region (OR 15.701, 95% CI 3.713–66.393, *p* < 0.001), and Region of the Americas (OR 8.605, 95% CI 2.256–32.819, *p* = 0.002) demonstrated significantly higher odds of PSC (Supplementary Table 6). Compared with high-middle SDI countries, studies conducted in low-middle SDI settings showed significantly lower odds of PSC (OR 0.144, 95% CI 0.036–0.572, *p* = 0.005) (Supplementary Table 6). Diagnosis based on ICD codes was associated with significantly lower reported PSC prevalence compared to studies using both imaging and biopsy (OR 0.211, 95% CI 0.068–0.651, *p* = 0.007) (Supplementary Table 6). Small-duct PSC had a significantly lower prevalence in UC compared to large-duct PSC (OR 0.192, 95% CI 0.063–0.588, *p* = 0.004) (Supplementary Table 6). Additionally, studies conducted before 2018 reported significantly higher odds of PSC than those published from 2018 onwards (OR 2.141, 95% CI 1.091–4.205, *p* = 0.027) (Supplementary Table 6).

Among patients with Crohn’s disease, compared to South-East Asian Region, only studies from the European Region reported significantly higher odds of PSC (OR 8.299, 95% CI 1.289–53.412, *p* = 0.026) (Supplementary Table 7). Compared to high-middle SDI countries, low-middle SDI countries had lower odds of PSC (OR 0.127, 95% CI 0.017–0.931, *p* = 0.042) (Supplementary Table 7). Again, diagnosis based on ICD codes was associated with significantly lower reported PSC prevalence compared to studies using both imaging and biopsy (OR 0.212, 95% CI 0.057–0.791, *p* = 0.021) (Supplementary Table 7). Studies conducted before 2018 also had significantly higher odds of PSC compared to studies published from 2018 onwards (OR 2.412, 95% CI 1.045–5.569, *p* = 0.039) (Supplementary Table 7).

### Sensitivity analyses

Leave-one-out sensitivity analyses demonstrated that omission of any single study did not materially alter the pooled prevalence estimates across all populations: general population (range: 7.24–9.93 per 100,000), IBD (14.3–16.1 per 1,000), ulcerative colitis (19.7–21.6 per 1,000), and Crohn's disease (9.3–10.9 per 1,000), supporting the robustness of findings despite high between-study heterogeneity (Supplementary Material 4). Similarly, leave-one-out analysis of incidence estimates yielded a narrow range of 0.84–0.95 per 100,000 person-years (Supplementary Material 4). Sensitivity analyses excluding ICD-only studies and pre-2018 administrative database studies yielded comparable pooled estimates, suggesting that the overall pooled estimates are not materially driven by any single ascertainment approach (Table 1, Table 2, Supplementary Table 3, Supplementary Table 4). Funnel plots for the main pooled analyses (general population prevalence and incidence, and prevalence in IBD, UC, and Crohn’s disease) are presented in Supplementary Material 5 for visual transparency. Restriction to high-quality studies (JBI score ≥ 80%) similarly did not materially alter pooled estimates, supporting the robustness of the primary findings (Table 1, Table 2, Supplementary Table 3, Supplementary Table 4).

## Discussion

In this systematic review and meta-analysis, we provide a comprehensive, updated synthesis of PSC epidemiology, estimating PSC prevalence in the general and IBD populations, and PSC incidence in the general population. The pooled overall prevalence of PSC in the general population was 8.06 per 100,000, while within IBD, PSC prevalence was substantially higher at 15.2 per 1,000. In both IBD and general populations, large-duct PSC remains the predominant subtype with a prevalence of 32.2 per 1,000 and 11.1 per 100,000, compared to 5.0 per 1,000 and 1.24 per 100,000. Heterogeneity was high for all outcomes, and this warrants emphasis: the wide prediction intervals spanning 0.57–57.34 per 100,000 in the general population and 1.9–113.4 per 1,000 in IBD indicate that the pooled values represent a best central estimate rather than a figure expected to hold in any individual population. Subgroup and regional estimates should therefore be regarded as the primary basis for clinical and policy interpretation. Collectively, these results reinforce PSC as a rare disease in the general population but a clinically important comorbidity in IBD, while highlighting persistent challenges in cross-study comparability. Overall findings are concordant with prior systematic reviews on this topic but add to the literature by providing the most granular subgroup characterization to date, examining relations to geography, socioeconomic context, time period, IBD phenotype, PSC subtype, diagnostic modality, and case-ascertainment method [8, 18, 19].

Overall, PSC prevalence was greater in UC than in Crohn’s disease. The highest prevalence observed in ileocolonic Crohn’s disease may reflect misclassification and overlap with the PSC-UC phenotype, which often demonstrates right-sided colitis with terminal ileal involvement that does not map cleanly onto classical Crohn’s disease categories. In particular, UC with pancolitis had the highest prevalence of PSC while prevalence was much lower in left-sided disease or proctitis. Notably, this contrasts with the classical clinical phenotype of PSC-associated colitis, typically characterized by findings of milder right-sided colitis. [20]. More extensive colitis is postulated to amplify enterohepatic immune trafficking, mucosal permeability, and microbial translocation [21], increasing the probability of cholangiopathy compared with limited distal disease or isolated ileal disease. In contrast, there was no statistically significant difference in PSC prevalence based on Crohn’s disease distribution due to wide confidence intervals and a small number of cases. Notably, the prevalence of small-duct PSC in Crohn’s disease was greater than that of small-duct PSC in UC (15.5 vs. 3.0 per 1,000 respectively). It may be worth noting the controversy surrounding the entity of small-duct PSC, with some evidence suggesting it may represent an early or *forme fruste* of large-duct PSC, as a substantial proportion of patients progress to classical large-duct disease over time [22]. The clinical implications of these findings are that clinicians should maintain a high index of suspicion for PSC in patients with IBD who have otherwise unexplained cholestasis and have a low threshold to assess for biliary strictures. While liver biopsy is often appropriate to assess for small-duct PSC in this context, this may be even more true in Crohn’s disease with cholestatic laboratory findings, where a comprehensive work-up of unexplained cholestasis should include assessment for alternative and overlapping etiologies, including available cholestatic genetic testing.

Since 2018, the prevalence of PSC in the general population has increased while prevalence in patients with IBD has shown a non-significant trend toward decrease (*p* = 0.335). The apparent increase in PSC prevalence in the general population may partly reflect the massive increase in incidence of IBD (the primary risk factor for PSC) in recent years, especially in recently industrialized countries. [23]. Why PSC prevalence among patients with IBD would have decreased over time is less clear. Although speculative and not supported by statistically significant temporal differences in the present analysis, one hypothesis worthy of future investigation is whether more aggressive IBD therapy in recent years, with the availability of drugs targeting mechanisms other than TNF-alpha such as vedolizumab (first approved in 2014), ustekinumab (2016), and tofacitinib (2018), as well as the shift from a primary objective of clinical remission [24, 25] to endoscopic or even histologic remission, may have influenced PSC prevalence in IBD populations [26, 27]. Studies on the effects of pharmacotherapy on PSC have yielded mixed results; however, colectomy prior to, but not after, a diagnosis of PSC has been associated with reduced need for liver transplant, implying that IBD disease control in the earliest stages of PSC pathogenesis may be protective [28, 29].

We observed pronounced regional variability in PSC prevalence across both the general population and IBD populations, and we emphasize that regional subgroup estimates should be the primary basis for clinical and policy interpretation rather than the overall pooled values alone. This is in line with known medical literature which postulates that the complex interplay between HLA risk haplotype, region-specific gut microbiome profiles shaped by diet and early-life exposures as well as gene–environment interactions influence this regional variation [30–32]. Importantly, the paucity of population-based data from the African Region likely contributes to the observed heterogeneity as well and may limit the precision and generalizability of regional prevalence estimates [33]. It should be noted that the present synthesis predominantly draws on data from European, North American, and high-SDI settings, and findings may not be directly generalizable to underrepresented regions, including Africa, South-East Asia, and low-to-middle SDI countries. Lower prevalence estimates based on meta-regression were consistently associated with lower-middle socio-demographic strata in the general IBD population (OR 0.16), as well as in the individual UC (OR 0.14) and Crohn’s disease cohorts (OR 0.13) vs. higher socio-demographic strata. Health-system and case-identification factors are also likely contributors: studies relying on administrative coding tended to produce lower prevalence estimates than clinically/imaging-defined cohorts. Similarly, administrative database ascertainment produced lower incidence estimates than hospital and clinic-based designs (0.60 vs. 1.06 per 100,000 person-years). Taken together, these findings suggest that observed regional differences in PSC prevalence may be driven by both the influences of geography on disease biology and evolving diagnostic pathways, accuracy of diagnostic codes, and study methodology, reinforcing that the overall pooled estimate should be interpreted with caution and contextualized within the specific regional and methodological setting of each included study. Finally, overall survival outcomes in PSC, including the prognostic impact of concomitant IBD, cholangiocarcinoma, and liver transplantation status, represent important priorities for future investigation beyond the scope of the present epidemiological synthesis.

### Limitations

This review was not prospectively registered with *PROSPERO*; however, the review was conducted and reported in accordance with the PRISMA guidelines to ensure methodological rigor. Formal publication bias assessment via funnel plots or Egger’s test was not performed; under conditions of extreme heterogeneity, funnel plot asymmetry cannot be reliably distinguished from between-study variance, rendering such assessments uninformative in this context [34]. Funnel plots for the main pooled analyses are nonetheless presented for visual transparency in Supplementary Material 5. Several additional limitations warrant consideration. Foremost is the very high heterogeneity across almost all pooled estimates, limiting the precision and generalizability of single summary values. Wide prediction intervals across all pooled estimates further contextualize this uncertainty, indicating that while the pooled estimates provide a best central estimate, true PSC prevalence in any individual population may deviate substantially from this value. It is worth noting that overall pooled values obscure substantial between-region variation, and interpretation should prioritize regional and methodological context where available. Differences in study design, case definitions, and diagnostic modalities also likely introduce misclassification and selection bias, particularly for small-duct PSC and for datasets reliant on ICD coding. Clinicians and researchers seeking methodology-specific estimates should therefore refer primarily to subgroup analyses stratified by diagnostic modality and case-ascertainment method, rather than the overall pooled values. Additionally, interpretation of analyses involving small-duct PSC should be undertaken cautiously given the limited number of available studies, relatively small sample sizes, and substantial between-study heterogeneity, which may reduce the precision and reliability of pooled estimates. As such, future work should focus on standardizing case definitions with explicit reporting of diagnostic criteria and PSC phenotypes, implementation of validated case-finding protocols, and development of prospective, population-based registries in underrepresented regions—particularly in lower-middle SDI. Additionally, restricting the search to MEDLINE (Ovid) and Embase may have contributed to the under-capture of regional literature and gray literature, particularly from these underrepresented regions. Future updates to this review should therefore consider broader database coverage. In parallel, consistent reporting of IBD subtypes and colitis extent is essential to enable clinically meaningful stratification and cross-study comparability to better our understanding of the influence of IBD on PSC prevalence. Furthermore, IBD cohorts likely differed in intensity of liver biochemistry monitoring and indications for cholangiography evaluation, creating detection bias. Subclinical or pre-clinical PSC may also be under-recognized, and incidental detection on imaging performed for other indications (e.g., magnetic resonance enterography) may inflate ascertainment in some settings while remaining undetected in others. Lastly, temporal comparisons involving administrative coding should be interpreted cautiously given the introduction of the dedicated PSC ICD-10-CM code (K83.01) in 2018, which may contribute to apparent increases in prevalence in more recent database studies.

## Conclusion

PSC remains a rare disease in the general population but a substantial comorbidity in IBD, especially in patients with UC pancolitis. The observed variability may be driven by both disease biology (e.g., effects of diet and lifestyle on the microbiota) and ascertainment effects related to health-system capacity, study design, and reliance on administrative coding. These data provide an epidemiological foundation for clinical planning and future studies on risk stratification, early detection, and PSC surveillance, and underscore the need for standardized, validated, and globally inclusive epidemiological data, particularly from underrepresented lower-SDI regions.

## Supplementary Information

Below is the link to the electronic supplementary material.

Supplementary file1 (DOCX 26 KB)

Supplementary file2 (PDF 925 KB)

Supplementary file3 (PDF 1215 KB)

Supplementary file4 (DOCX 36 KB)

Supplementary file5 (PDF 1580 KB)

Supplementary file6 (PDF 1547 KB)

Supplementary file7 (DOCX 101 KB)

## Data Availability

All articles in this manuscript are available from MEDLINE (Ovid) and EMBASE. The data extraction sheets and statistical analysis code used in this study are available from the corresponding author upon reasonable request.

## Abbreviations

CD: Crohn’s disease
ERCP: Endoscopic retrograde cholangiopancreatography
IBD: Inflammatory bowel disease
ICD: International classification of diseases
IHME: Institute of Health Metrics and Evaluation
IRB: Institutional Review Board
JBI: Joanna Briggs Institute
MRCP: Magnetic resonance cholangiopancreatography
PSC: Primary sclerosing cholangitis
REML: Restricted maximum likelihood
SDI: Socio-demographic index
UC: Ulcerative colitis
WHO: World Health Organization
