# Supplementary material for: Epidemiology of primary sclerosing cholangitis in general and IBD populations: a systematic review and meta-analysis
Source: Hepatol Int. 2026 Jul 11;20(5):1463–78. doi: 10.1007/s12072-026-11127-y (PMC13676655; doi:10.1007/s12072-026-11127-y)

Supplementary Material 2. Prevalence of primary sclerosing cholangitis and subtypes within the general population (per 100,000)

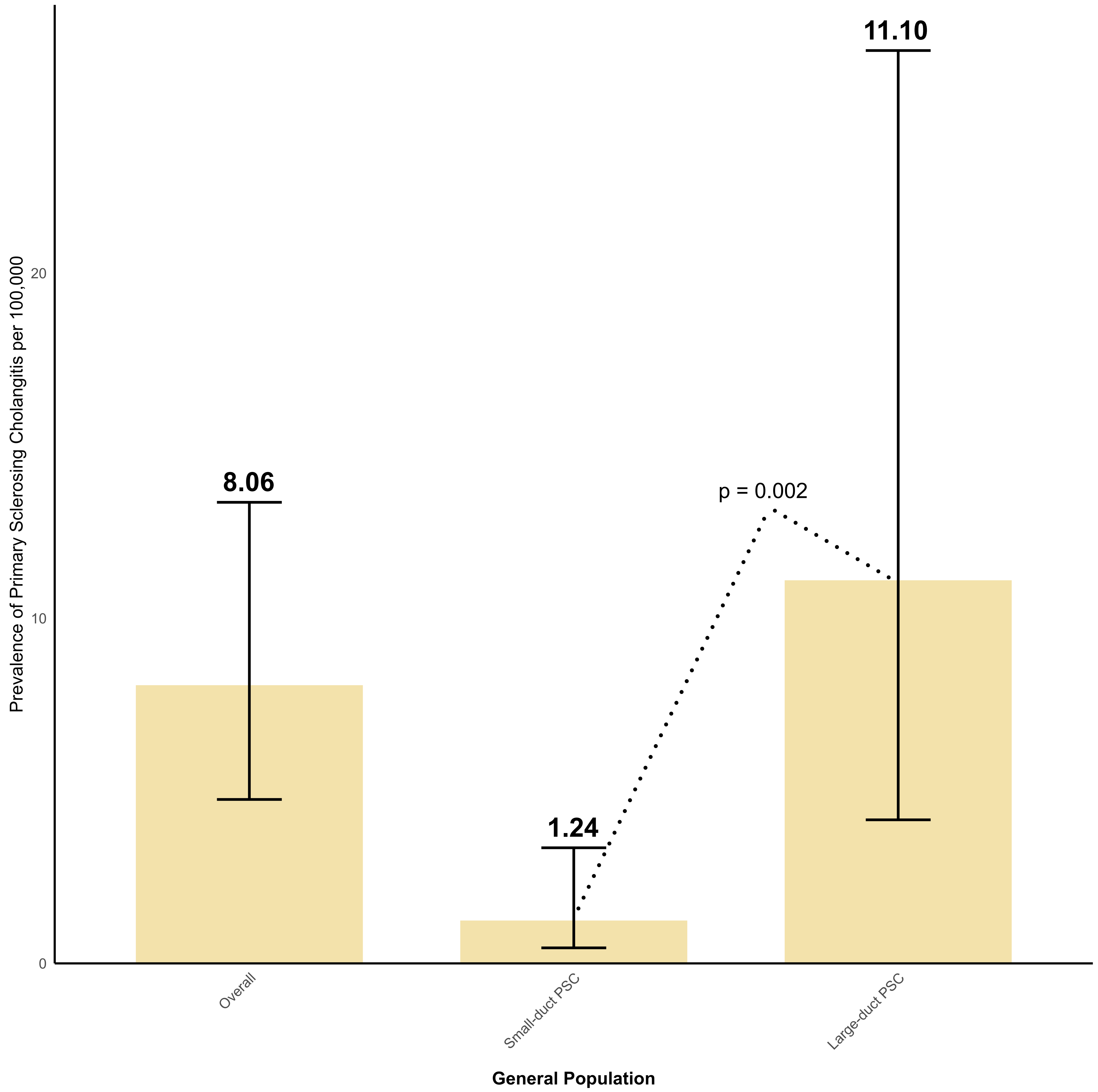

Supplement: Supplementary file 1 [file 12072_2026_11127_MOESM2_ESM.pdf]
