# Supplementary material for: Epidemiology of primary sclerosing cholangitis in general and IBD populations: a systematic review and meta-analysis
Source: Hepatol Int. 2026 Jul 11;20(5):1463–78. doi: 10.1007/s12072-026-11127-y (PMC13676655; doi:10.1007/s12072-026-11127-y)

Supplementary Material 3. Forest plot of pooled incidence rates (per 100,000 person-years) of primary sclerosing cholangitis in the general population and subgroup analyses

Subgroup	IR	95% CI	p value
Overall	0.892	[0.705; 1.130]	
**WHO Region**			p = 0.683
European Region	0.889	[0.662; 1.190]	
Region of the Americas	0.792	[0.412; 1.525]	
Western Pacific Region	1.113	[0.659; 1.880]	
**Socio-Demographic Index**			p < 0.001
High	0.892	[0.705; 1.130]	
**Dates**			p = 0.429
Before 2018	0.839	[0.601; 1.172]	
2018 Onwards	1.008	[0.740; 1.372]	
**Case-Ascertainment Method**			p = 0.004
Administrative database studies	0.599	[0.448; 0.802]	
Hospital/ Clinic-based Studies	1.057	[0.824; 1.356]	
**Diagnosis Method**			p < 0.001
Imaging	1.314	[1.051; 1.643]	
Both Imaging and Biopsy	0.823	[0.583; 1.163]	
Others/ Unspecified	0.689	[0.596; 0.798]	

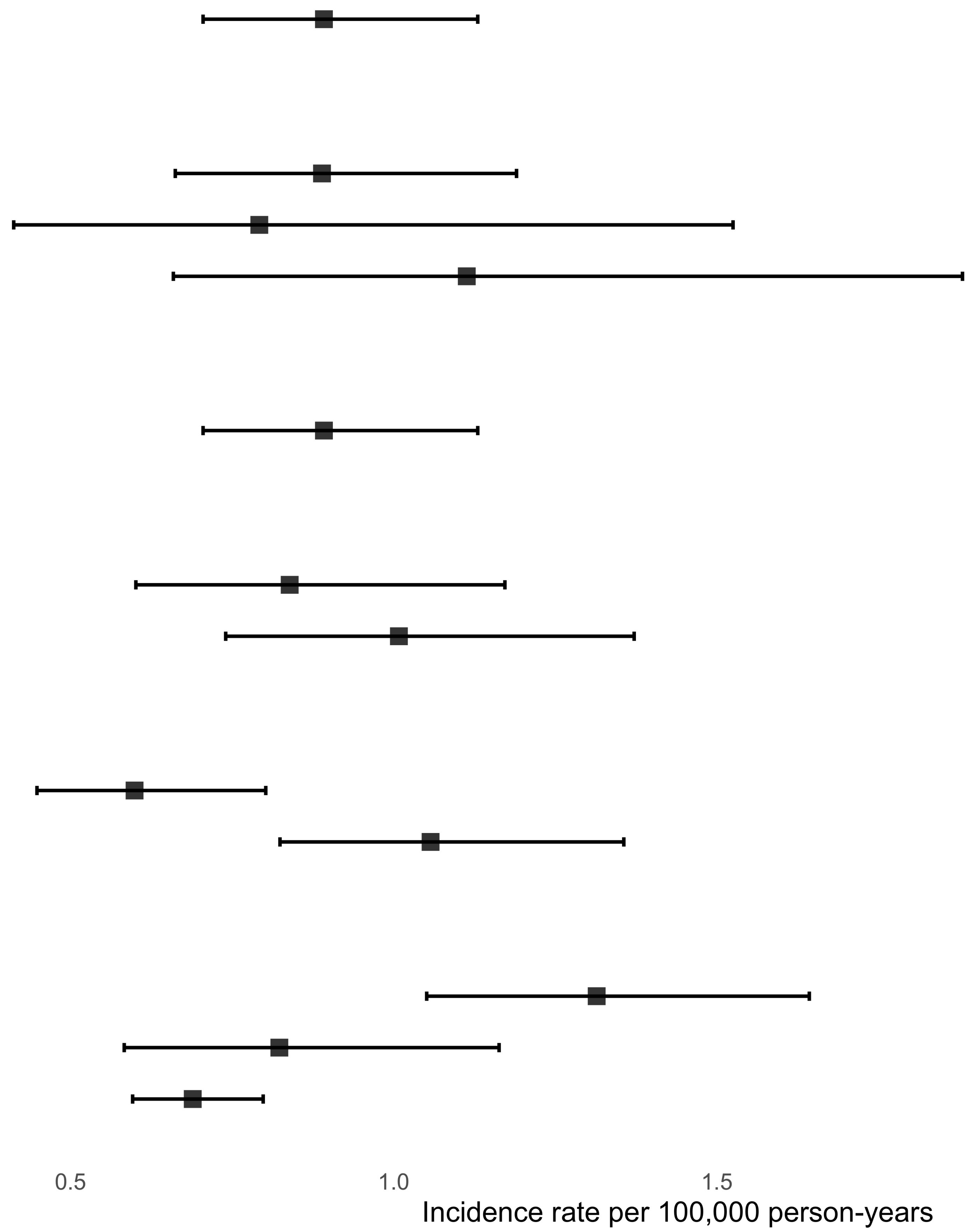

Supplement: Supplementary file 2 [file 12072_2026_11127_MOESM3_ESM.pdf]
