# Supplementary material for: Epidemiology of primary sclerosing cholangitis in general and IBD populations: a systematic review and meta-analysis
Source: Hepatol Int. 2026 Jul 11;20(5):1463–78. doi: 10.1007/s12072-026-11127-y (PMC13676655; doi:10.1007/s12072-026-11127-y)
Supplement: Supplementary file 3 [file 12072_2026_11127_MOESM5_ESM.pdf]

Supplementary Material 4. Leave-one-out sensitivity analysis forest plots for the pooled prevalence of primary sclerosing cholangitis (PSC) in: (A) general population; (B) inflammatory bowel disease (IBD) population; (C) ulcerative colitis (UC) population; (D) Crohn's disease (CD) population; and (E) incidence rate in the general population

A

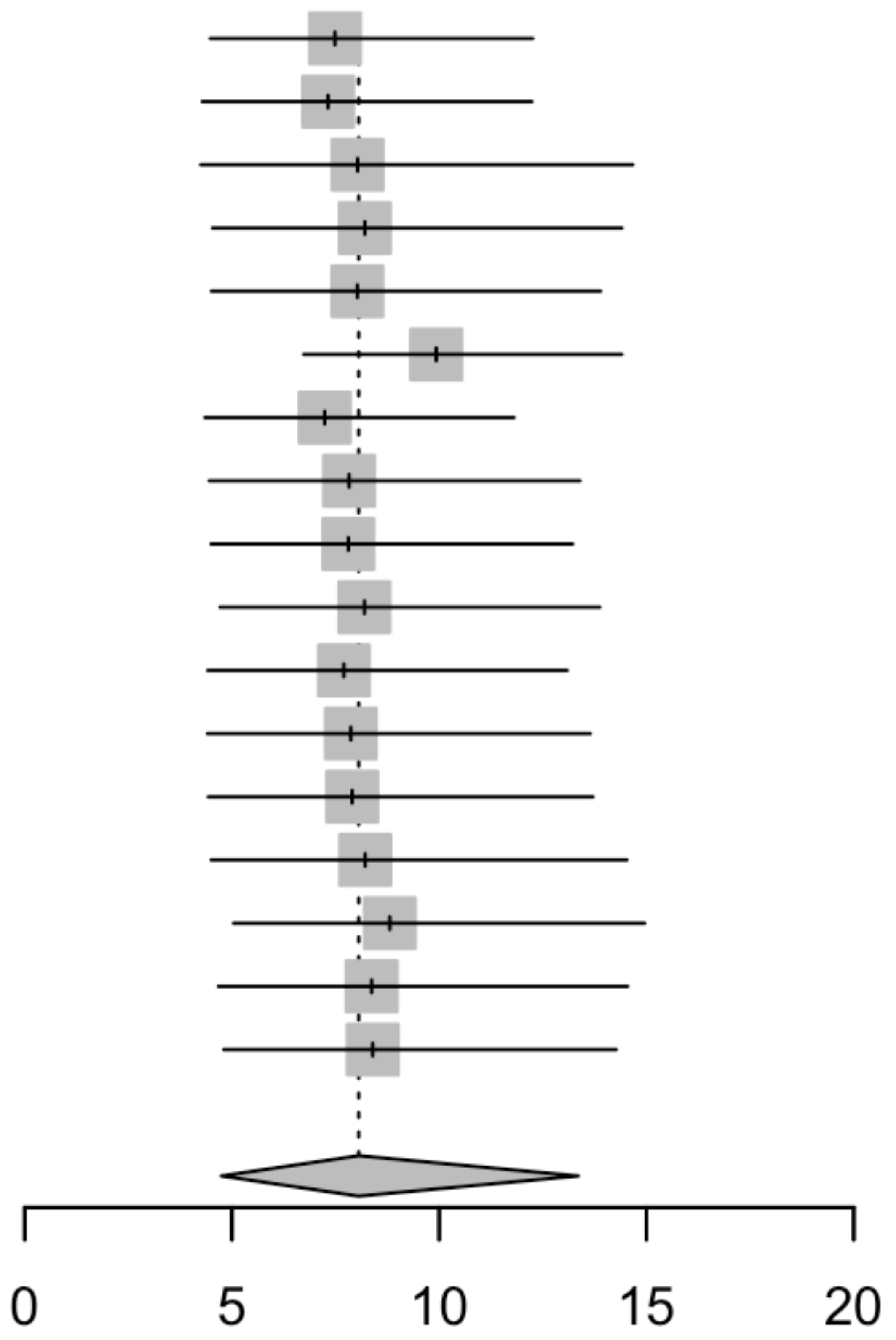

Study	Prevalence (per 100,000)	95% CI	I²
Omitting Bakhshi et al.	7.49	[4.48; 12.26]	100%
Omitting Barner-Rasmussen et al.	7.32	[4.28; 12.24]	100%
Omitting Boberg et al.	8.03	[4.25; 14.67]	100%
Omitting Boonstra et al.	8.21	[4.53; 14.41]	100%
Omitting Dyson et al.	8.03	[4.51; 13.89]	100%
Omitting Escorsell et al.	9.93	[6.73; 14.41]	100%
Omitting Fernandez et al.	7.24	[4.35; 11.81]	100%
Omitting Kingham et al.	7.83	[4.45; 13.40]	100%
Omitting Lamba et al.	7.81	[4.50; 13.22]	100%
Omitting Liang et al.	8.20	[4.72; 13.87]	100%
Omitting Lindkvist et al.	7.70	[4.42; 13.09]	100%
Omitting Ngu et al.	7.87	[4.41; 13.65]	100%
Omitting Nielsen et al.	7.90	[4.43; 13.71]	100%
Omitting Tan et al.	8.22	[4.50; 14.52]	100%
Omitting Tanaka et al.	8.81	[5.04; 14.95]	100%
Omitting Toy et al.	8.37	[4.67; 14.54]	100%
Omitting Webb et al.	8.40	[4.81; 14.27]	100%
**Random effects model**	**8.06**	**[4.75; 13.36]**	**100%**

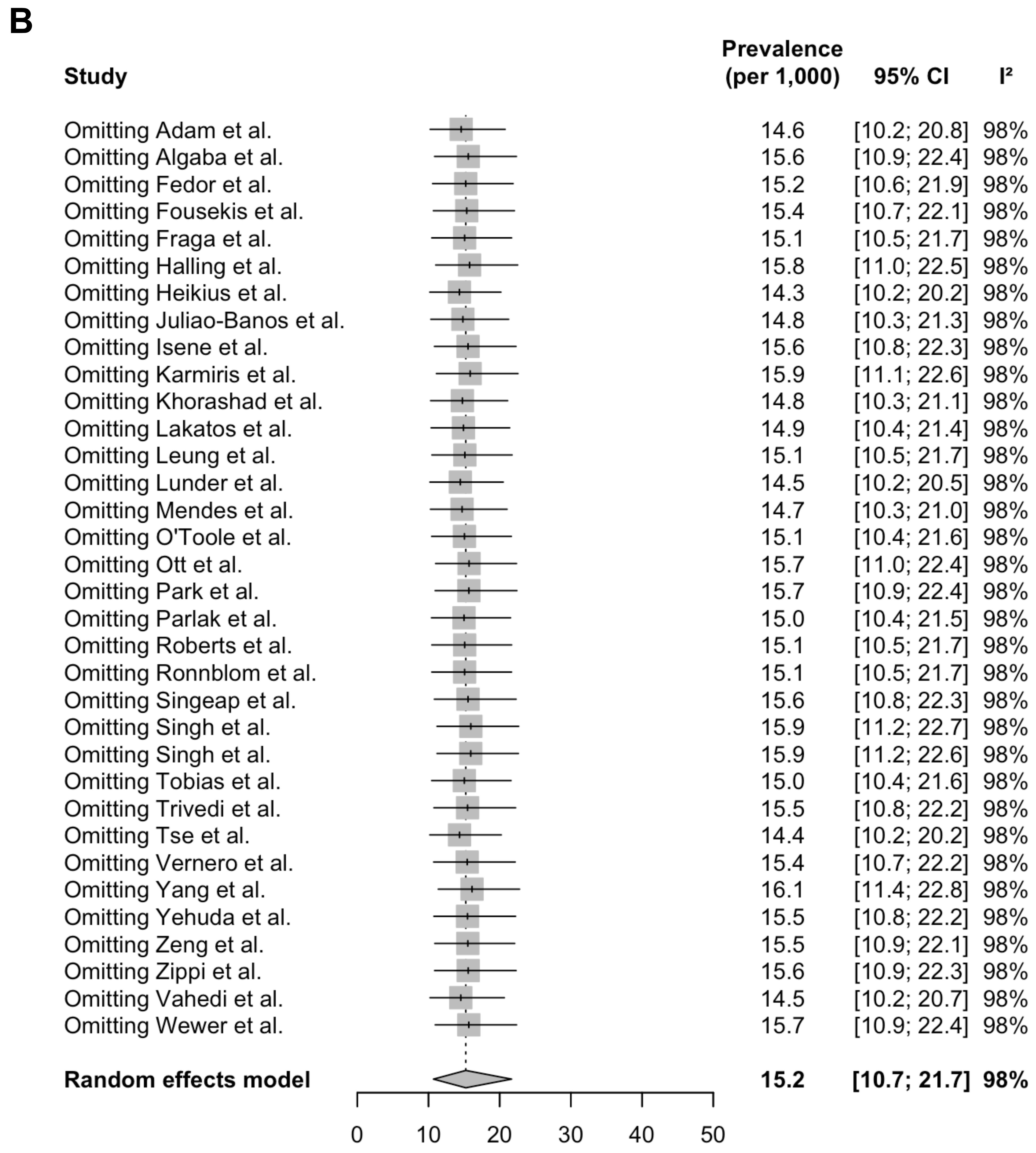
B
Study
Prevalence (per 1,000)
95% CI
I²
Omitting Adam et al. 14.6 [10.2; 20.8] 98%
Omitting Algaba et al. 15.6 [10.9; 22.4] 98%
Omitting Fedor et al. 15.2 [10.6; 21.9] 98%
Omitting Fousekis et al. 15.4 [10.7; 22.1] 98%
Omitting Fraga et al. 15.1 [10.5; 21.7] 98%
Omitting Halling et al. 15.8 [11.0; 22.5] 98%
Omitting Heikius et al. 14.3 [10.2; 20.2] 98%
Omitting Juliao-Banos et al. 14.8 [10.3; 21.3] 98%
Omitting Isene et al. 15.6 [10.8; 22.3] 98%
Omitting Karmiris et al. 15.9 [11.1; 22.6] 98%
Omitting Khorashad et al. 14.8 [10.3; 21.1] 98%
Omitting Lakatos et al. 14.9 [10.4; 21.4] 98%
Omitting Leung et al. 15.1 [10.5; 21.7] 98%
Omitting Lunder et al. 14.5 [10.2; 20.5] 98%
Omitting Mendes et al. 14.7 [10.3; 21.0] 98%
Omitting O'Toole et al. 15.1 [10.4; 21.6] 98%
Omitting Ott et al. 15.7 [11.0; 22.4] 98%
Omitting Park et al. 15.7 [10.9; 22.4] 98%
Omitting Parlak et al. 15.0 [10.4; 21.5] 98%
Omitting Roberts et al. 15.1 [10.5; 21.7] 98%
Omitting Ronnblom et al. 15.1 [10.5; 21.7] 98%
Omitting Singeap et al. 15.6 [10.8; 22.3] 98%
Omitting Singh et al. 15.9 [11.2; 22.7] 98%
Omitting Singh et al. 15.9 [11.2; 22.6] 98%
Omitting Tobias et al. 15.0 [10.4; 21.6] 98%
Omitting Trivedi et al. 15.5 [10.8; 22.2] 98%
Omitting Tse et al. 14.4 [10.2; 20.2] 98%
Omitting Vernero et al. 15.4 [10.7; 22.2] 98%
Omitting Yang et al. 16.1 [11.4; 22.8] 98%
Omitting Yehuda et al. 15.5 [10.8; 22.2] 98%
Omitting Zeng et al. 15.5 [10.9; 22.1] 98%
Omitting Zippi et al. 15.6 [10.9; 22.3] 98%
Omitting Vahedi et al. 14.5 [10.2; 20.7] 98%
Omitting Wewer et al. 15.7 [10.9; 22.4] 98%
Random effects model 15.2 [10.7; 21.7] 98%
0
10
20
30
40
50

C

Study	Prevalence (per 1,000)	95% CI	I²
Omitting Adam et al.	19.8	[14.6; 26.7]	97%
Omitting Broome et al.	20.5	[15.0; 27.9]	97%
Omitting Fedor et al.	20.6	[15.1; 28.1]	97%
Omitting Fousekis et al.	21.1	[15.5; 28.6]	97%
Omitting Fraga et al.	20.2	[14.8; 27.5]	97%
Omitting Halling et al.	21.2	[15.6; 28.7]	96%
Omitting Hashimoto et al.	20.4	[14.9; 27.8]	97%
Omitting Heikius et al.	20.0	[14.7; 27.1]	97%
Omitting Juliao-Banos et al.	20.2	[14.8; 27.5]	97%
Omitting Isene et al.	21.3	[15.8; 28.8]	97%
Omitting Karmiris et al.	21.4	[15.8; 28.8]	97%
Omitting Khorashad et al.	20.1	[14.8; 27.4]	97%
Omitting Kumagai et al.	20.4	[14.9; 27.8]	97%
Omitting Lakatos et al.	20.4	[14.9; 27.8]	97%
Omitting Lepisto et al.	19.7	[14.6; 26.6]	96%
Omitting Lunder et al.	20.0	[14.7; 27.2]	97%
Omitting Lupinetti et al.	20.9	[15.3; 28.3]	97%
Omitting Petri Aitola et al.	19.8	[14.6; 26.8]	97%
Omitting O'Toole et al.	20.4	[14.9; 27.8]	97%
Omitting Olsson et al.	20.3	[14.8; 27.6]	97%
Omitting Ott et al.	20.8	[15.3; 28.2]	97%
Omitting Park et al.	21.0	[15.5; 28.6]	97%
Omitting Parlak et al.	20.5	[15.0; 27.9]	97%
Omitting Rasmussen et al.	20.3	[14.9; 27.7]	97%
Omitting Rawal et al.	21.1	[15.5; 28.6]	97%
Omitting Roberts et al.	20.3	[14.9; 27.7]	97%
Omitting Ronnblom et al.	20.6	[15.1; 28.1]	97%
Omitting Schrumpf et al.	20.1	[14.8; 27.3]	97%
Omitting Shepherd et al.	20.5	[15.0; 27.9]	97%
Omitting Singeap et al.	21.0	[15.4; 28.5]	97%
Omitting Singh et al.	21.4	[15.8; 28.9]	96%
Omitting Singh et al.	21.3	[15.7; 28.8]	97%
Omitting Terg et al.	20.4	[14.9; 27.8]	97%
Omitting Tobias et al.	20.4	[14.9; 27.7]	97%
Omitting Wei et al.	20.7	[15.2; 28.2]	97%
Omitting Xu, W et al.	21.0	[15.4; 28.5]	97%
Omitting Yamamoto et al.	20.0	[14.7; 27.2]	97%
Omitting Yang et al.	21.6	[16.0; 29.1]	96%
Omitting Yazdanbod et al.	20.6	[15.1; 28.1]	97%
Omitting Ye et al.	20.8	[15.3; 28.4]	97%
Omitting Yehuda et al.	20.9	[15.3; 28.4]	97%
Omitting Zeng et al.	20.8	[15.3; 28.2]	97%
Omitting Zippi et al.	21.1	[15.5; 28.6]	97%
Omitting Holzmann et al.	20.1	[14.7; 27.3]	97%
Omitting Koutroubakis et al.	20.0	[14.7; 27.2]	97%
Omitting Vahedi et al.	19.8	[14.6; 26.8]	96%
Omitting Wewer et al.	21.0	[15.4; 28.5]	97%
**Random effects model**	**20.6**	**[15.2; 27.8]**	**97%**

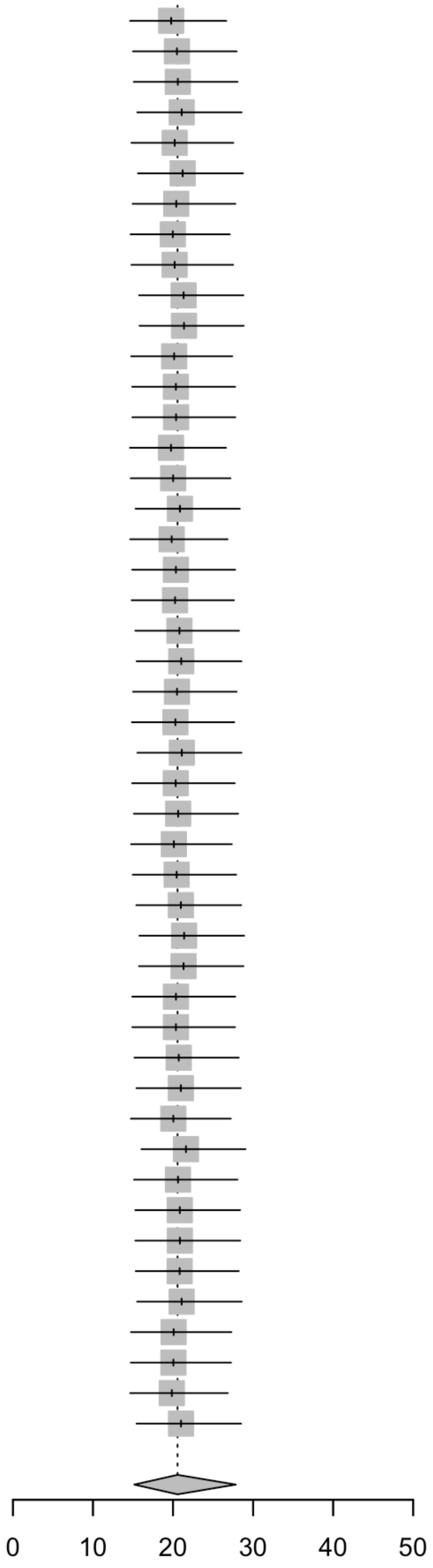

D

Study	Prevalence (per 1,000)	95% CI	I²
Omitting Adam et al.	10.3	[6.6; 16.1]	91%
Omitting Bruining et al.	10.0	[6.3; 15.8]	91%
Omitting Fedor et al.	10.1	[6.4; 15.9]	91%
Omitting Fousekis et al.	9.9	[6.3; 15.7]	91%
Omitting Fraga et al.	10.4	[6.6; 16.4]	91%
Omitting Halling et al.	10.8	[7.0; 16.8]	89%
Omitting Heikius et al.	9.3	[6.3; 13.9]	89%
Omitting Juliao-Banos et al.	10.1	[6.4; 15.9]	91%
Omitting Isene et al.	10.0	[6.3; 15.8]	91%
Omitting Karmiris et al.	10.5	[6.7; 16.4]	91%
Omitting Khorashad et al.	10.5	[6.7; 16.2]	91%
Omitting Lakatos et al.	9.8	[6.2; 15.3]	91%
Omitting Lunder et al.	9.4	[6.2; 14.2]	89%
Omitting O'Toole et al.	10.1	[6.4; 16.0]	91%
Omitting Ott et al.	10.6	[6.9; 16.5]	91%
Omitting Ozkahraman et al.	10.0	[6.4; 15.8]	91%
Omitting Park et al.	10.6	[6.8; 16.6]	91%
Omitting Parlak et al.	9.8	[6.2; 15.3]	91%
Omitting Rasmussen et al.	9.7	[6.2; 15.2]	91%
Omitting Roberts et al.	10.0	[6.3; 15.9]	91%
Omitting Ronnblom et al.	9.8	[6.2; 15.3]	91%
Omitting Singeap et al.	10.3	[6.5; 16.3]	91%
Omitting Singh et al.	10.9	[7.1; 16.9]	91%
Omitting Singh et al.	10.8	[7.0; 16.6]	91%
Omitting Tobias et al.	10.2	[6.4; 16.0]	91%
Omitting Yang et al.	10.9	[7.0; 16.9]	89%
Omitting Yehuda et al.	10.3	[6.5; 16.3]	91%
Omitting Zeng et al.	10.3	[6.6; 16.0]	91%
Omitting Zippi et al.	10.2	[6.5; 16.1]	91%
Omitting Vahedi et al.	9.8	[6.2; 15.4]	91%
Omitting Wewer et al.	10.6	[6.8; 16.4]	91%
**Random effects model**	**10.2**	**[6.6; 15.8]**	**91%**

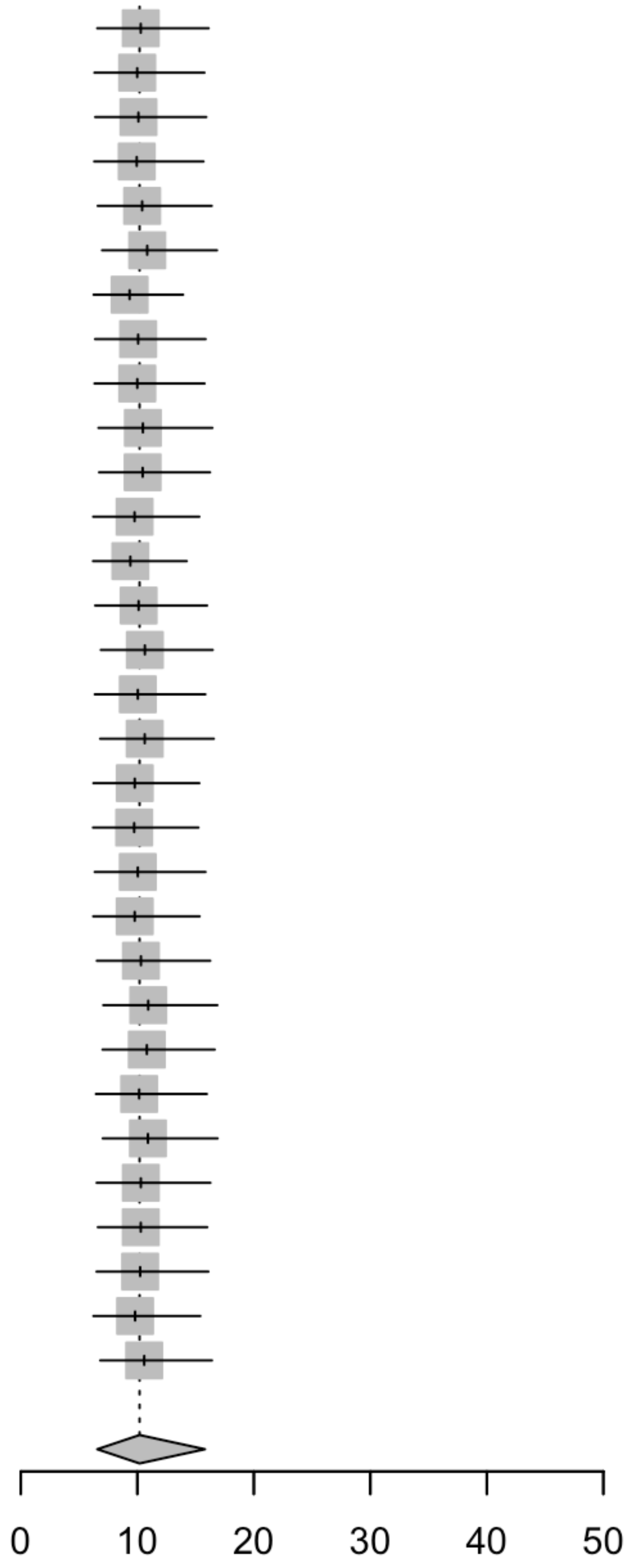

E

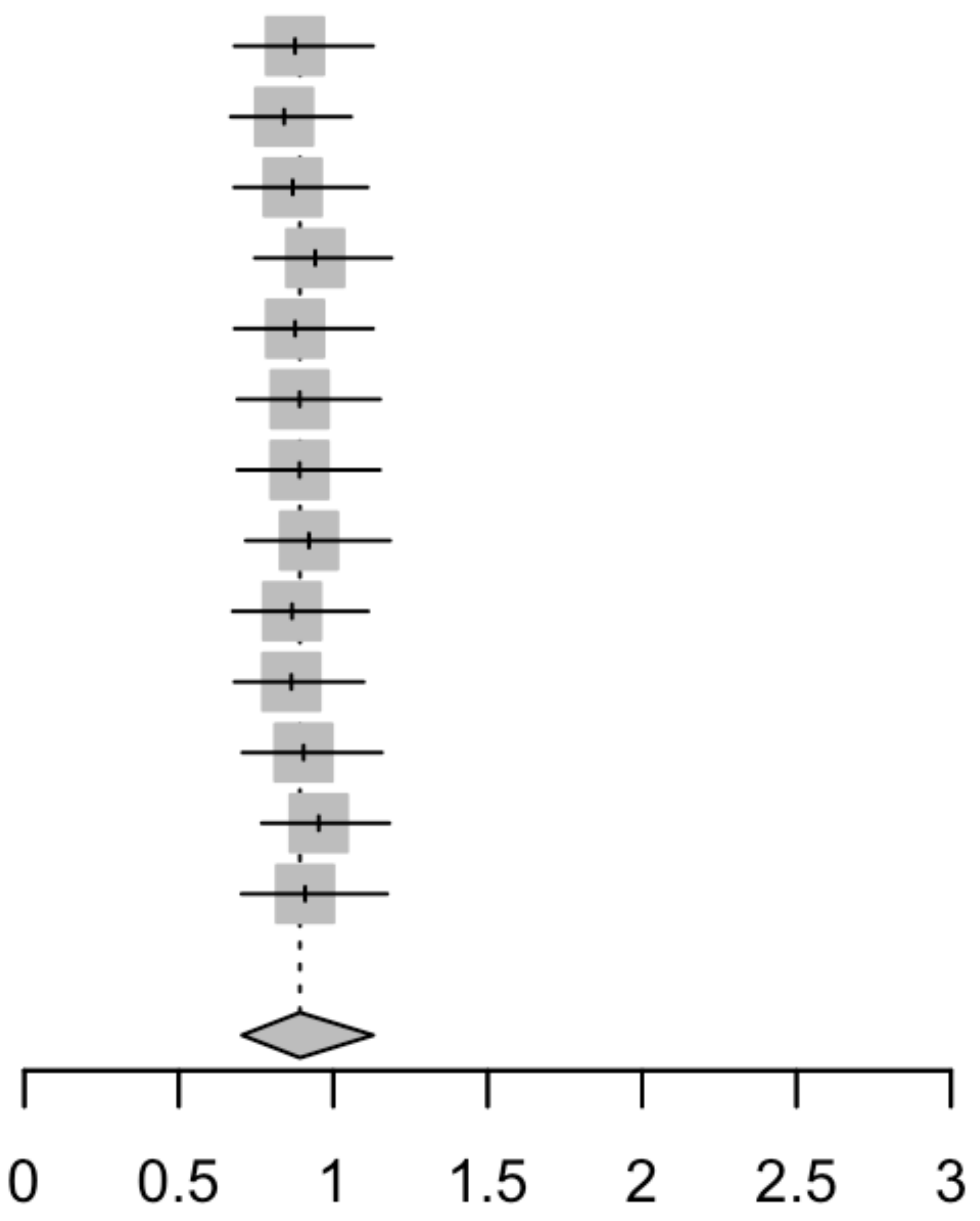

Study	Incidence Rate (per 100,000 PY)	95% CI	I²
Omitting Bakhshi	0.88	[0.68; 1.13]	97%
Omitting Barner-Rasmussen	0.84	[0.67; 1.06]	93%
Omitting Boberg	0.87	[0.68; 1.11]	97%
Omitting Boonstra	0.94	[0.75; 1.19]	97%
Omitting Kaplan	0.88	[0.68; 1.13]	97%
Omitting Kingham	0.89	[0.69; 1.15]	97%
Omitting Lamba	0.89	[0.69; 1.15]	97%
Omitting Liang	0.92	[0.72; 1.18]	97%
Omitting Lindkvist	0.87	[0.67; 1.11]	97%
Omitting Ngu	0.86	[0.68; 1.10]	97%
Omitting Nielsen	0.90	[0.71; 1.16]	97%
Omitting Toy	0.95	[0.77; 1.18]	97%
Omitting Webb	0.91	[0.70; 1.17]	97%
**Random effects model**	**0.89**	**[0.70; 1.13]**	**97%**
