# Supplementary material for: Epidemiology of primary sclerosing cholangitis in general and IBD populations: a systematic review and meta-analysis
Source: Hepatol Int. 2026 Jul 11;20(5):1463–78. doi: 10.1007/s12072-026-11127-y (PMC13676655; doi:10.1007/s12072-026-11127-y)
Supplement: Supplementary file 4 [file 12072_2026_11127_MOESM6_ESM.pdf]

Supplementary Material 5. Funnel plot for pooled prevalence of primary sclerosing cholangitis (PSC) in (A) general population; (B) inflammatory bowel disease (IBD population); (C) ulcerative colitis (UC) population; (D) Crohn's disease (CD) population; and (E) pooled incidence of PSC in the general population

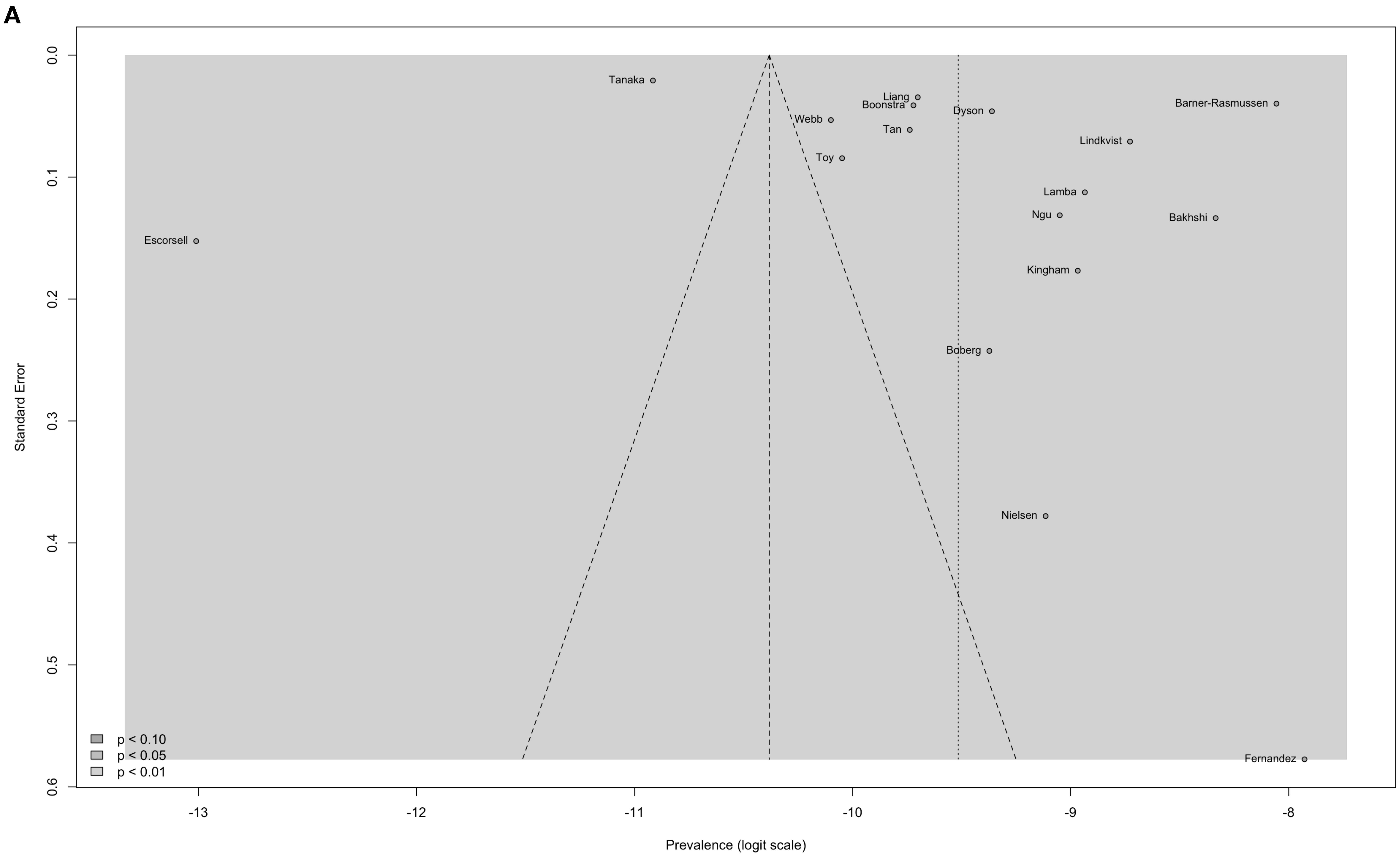

B

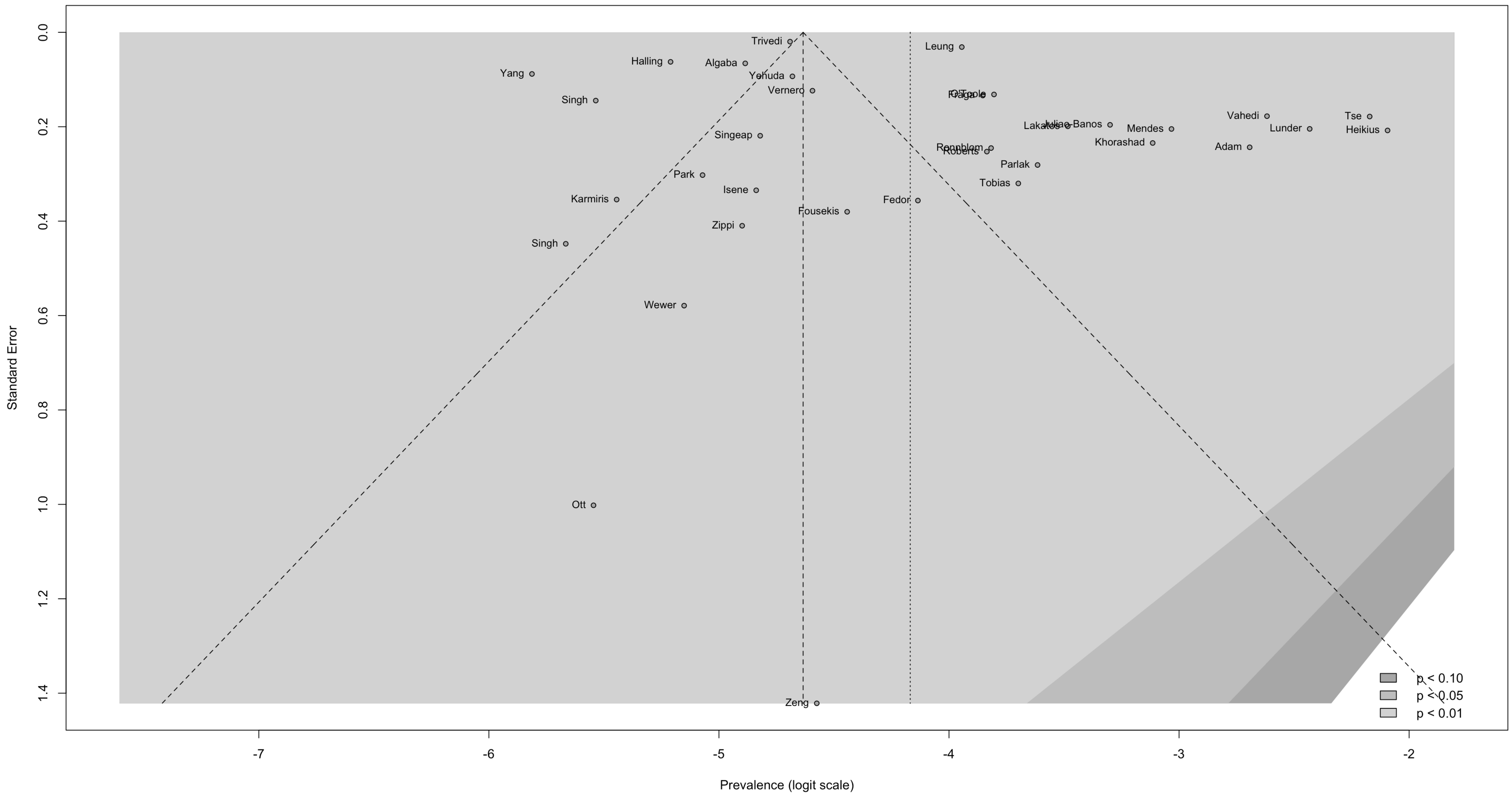
Standard Error
Prevalence (logit scale)
0.0
0.2
0.4
0.6
0.8
1.0
1.2
1.4
-7
-6
-5
-4
-3
-2
p < 0.10
p < 0.05
p < 0.01
Trivedi
Leung
Halling
Algaba
Yang
Yehuda
Vernero
Singh
O'Toole
Fraga
Vahedi
Tse
Lakatos
Julian-Banos
Mendes
Lunder
Heikius
Singeap
Khorashad
Adam
Ronnblom
Roberts
Parlak
Park
Isene
Tobias
Karmiris
Fedor
Fousekis
Zippi
Singh
Wewer
Ott
Zeng

C

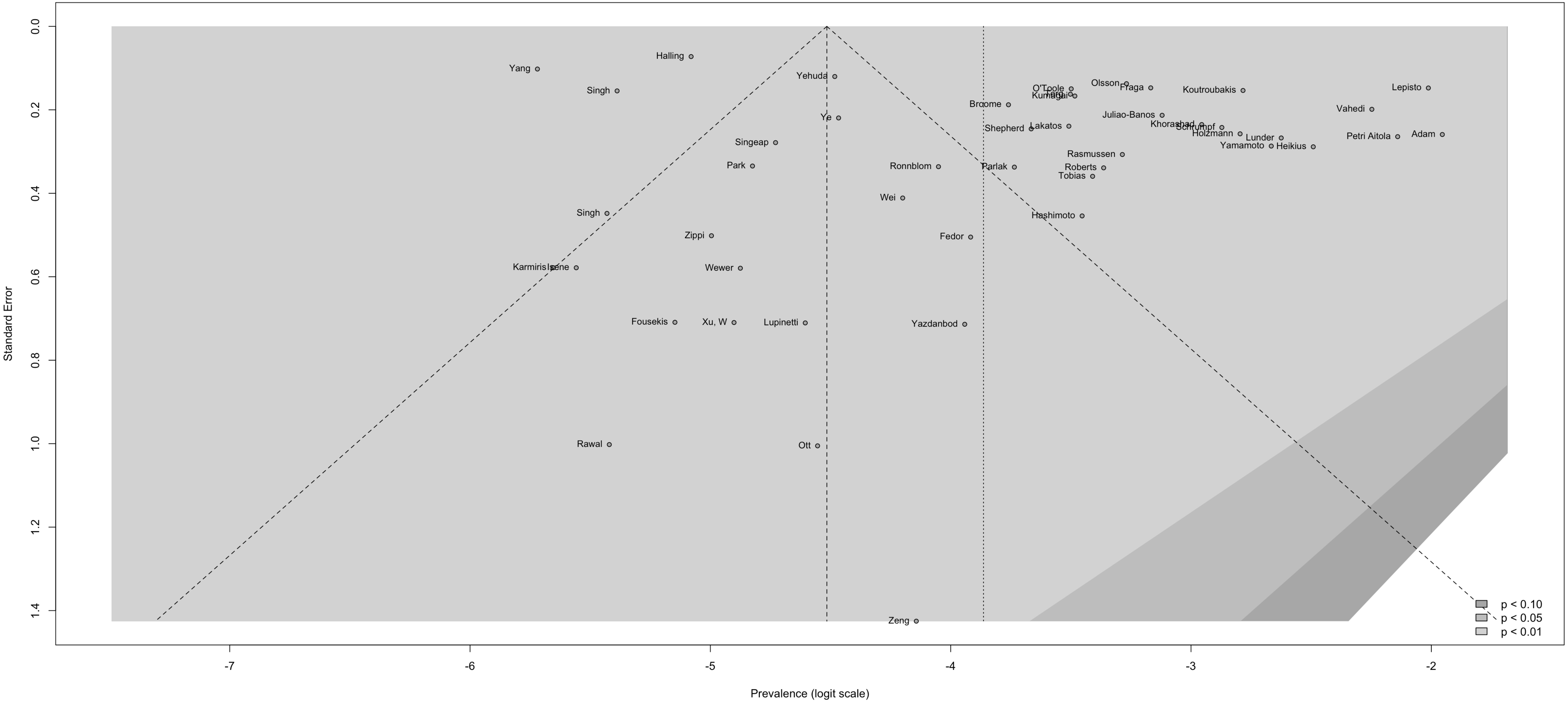
Standard Error
Prevalence (logit scale)
0.0
0.2
0.4
0.6
0.8
1.0
1.2
1.4
-7
-6
-5
-4
-3
-2
p < 0.10
p < 0.05
p < 0.01
Halling
Yang
Singh
Yehuda
Ye
Singeap
Park
Ronnblom
Parlak
Wei
Singh
Zippi
Fedor
Karmiris
Isene
Wewer
Fousekis
Xu, W
Lupinetti
Yazdanbod
Rawal
Ott
Zeng
Olsson
Fraga
Koutroubakis
Lepisto
Broome
Juliao-Banos
Vahedi
Shepherd
Lakatos
Lunder
Petri Aitola
Adam
Yamamoto
Heikius
Rasmussen
Roberts
Tobias
Hashimoto

D

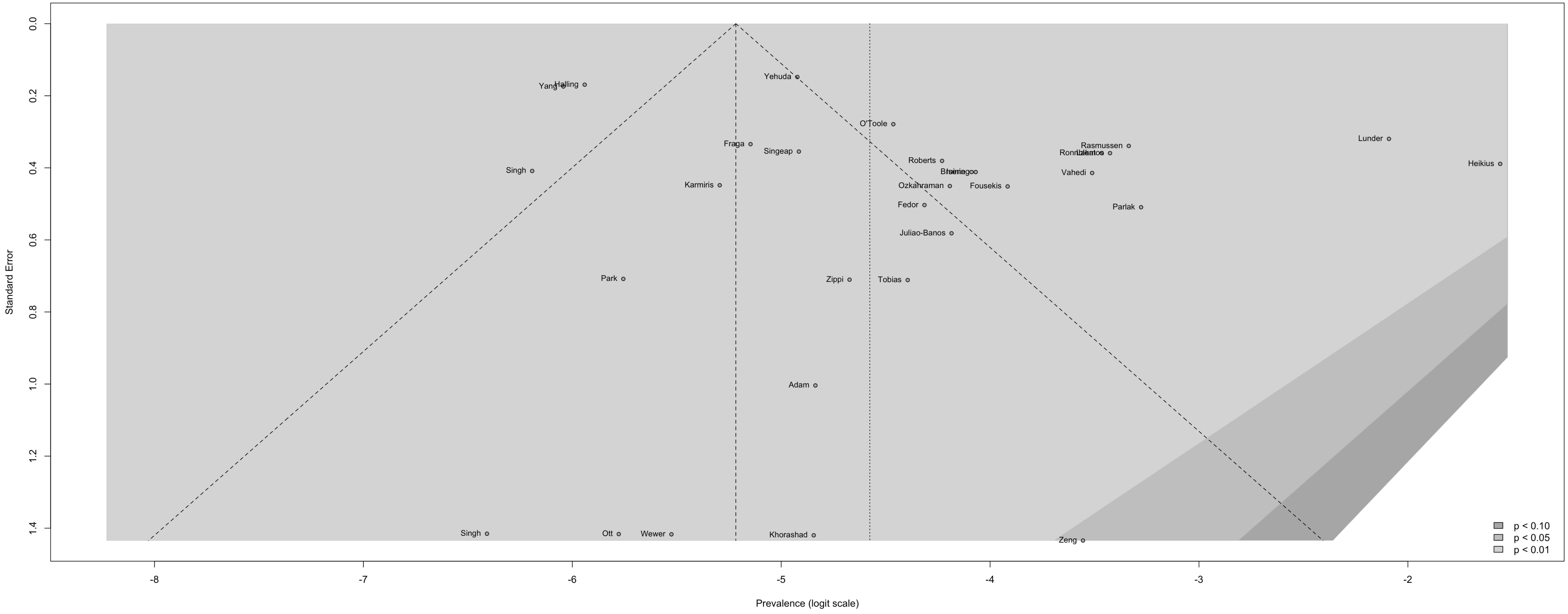
Standard Error
Prevalence (logit scale)
0.0
0.2
0.4
0.6
0.8
1.0
1.2
1.4
-8
-7
-6
-5
-4
-3
-2
Yehuda
Halling
Yang
O'Toole
Lunder
Fraga
Singeap
Rasmussen
Roberts
Heikius
Singh
Vahedi
Karmiris
Ozkahraman
Fousekis
Fedor
Parlak
Juliao-Banos
Park
Zippi
Tobias
Adam
Singh
Ott
Wewer
Khorashad
Zeng
p < 0.10
p < 0.05
p < 0.01

E

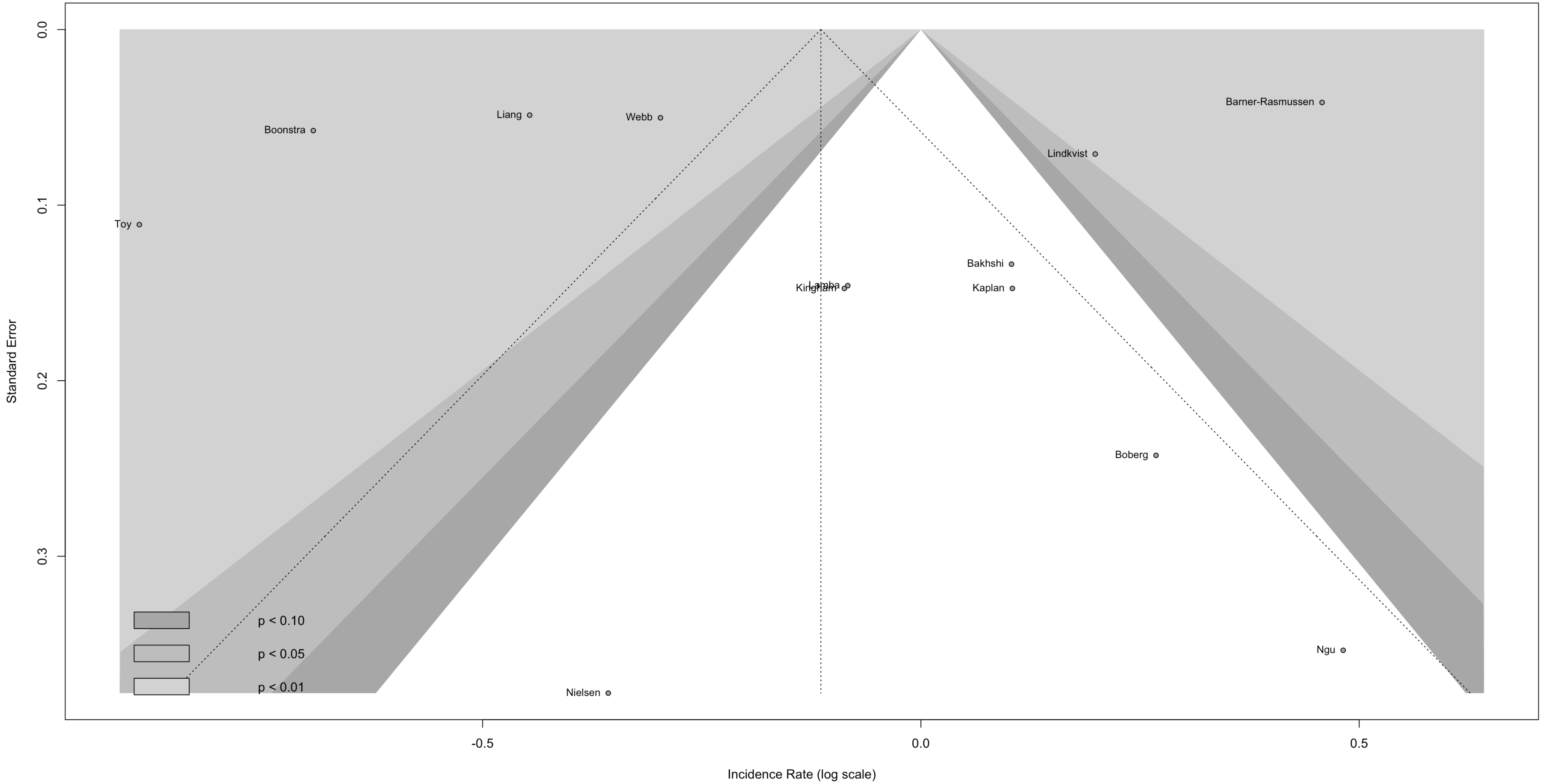

Standard Error
0.0
0.1
0.2
0.3
Barner-Rasmussen
Liang
Webb
Boonstra
Lindkvist
Toy
Bakhshi
Lamba
Kingham
Kaplan
Boberg
Ngu
Nielsen
p < 0.10
p < 0.05
p < 0.01
-0.5
0.0
0.5
Incidence Rate (log scale)
