# Supplementary material for: Epidemiology of primary sclerosing cholangitis in general and IBD populations: a systematic review and meta-analysis
Source: Hepatol Int. 2026 Jul 11;20(5):1463–78. doi: 10.1007/s12072-026-11127-y (PMC13676655; doi:10.1007/s12072-026-11127-y)
Supplement: Supplementary file 5 [file 12072_2026_11127_MOESM1_ESM.docx]

**Supplementary Material 1. Search Strategy**

**EMBASE VIA EMBASE.COM**

1. ‘primary sclerosing cholangitis’/exp or ‘sclerosing cholangitis’/exp or ‘primary sclerosing cholangitis’ or ‘autoimmune cholangitis’/exp or ‘autoimmune cholangitis’ or ‘autoimmune cholangiopathy’/exp or ‘autoimmune cholangiopathy’
2. (‘scleros* cholangiti*’ or ‘psc’ or 'fibros* cholangi*' or 'stenos* cholangi*' or 'obliterat* cholangi*' or 'autoimmune cholang*’ or ‘autoimmune bil*’ or ‘autoimmune cholestat*’ or ‘autoimmune liver disease*’ or ‘hepatobiliary adj5 manifestation*’):ti,ab,kw
3. 1 or 2
4. (‘epidemiology’ or ‘prevalence’ or ‘incidence’ or ‘mortality’ or ‘morbidity’ or ‘surveillance’)/exp
5. (‘epidemiolog*’ or ‘prevalen*’ or ‘inciden*’ or ‘mortal*’ or ‘morbidit*’ or ‘surveillan*’ or ‘registry’ or ‘database’ or ‘burden’ or ‘diagnos*’):ti,ab
6. 4 or 5
7. 3 and 6
8. 7 and ([article]/lim or [letter]/lim) and [english]/lim

**MEDLINE VIA OVID**

1. exp Cholangitis, Sclerosing/
2. (sclerosing cholangi* or psc or autoimmune cholangi* or autoimmune bil* or autoimmune cholestat* or fibros* cholangi* or stenos* cholangi* or obliterate* cholangi* or autoimmune liver disease or (hepatobiliary adj5 manifestation*)).ti,ab.
3. 1 or 2
4. exp Epidemiology/ or exp Prevalence/ or exp Incidence/ or exp Mortality/ or exp Morbidity/
5. (epidemiolog* or prevalen* or incidenc* or mortalit* or morbidit* or surveillan* or registry or database or burden or diagnos*).ti,ab.
6. 4 or 5
7. 3 and 6
8. limit 7 to (english language and journal article)
