# Supplementary material for: Epidemiology of primary sclerosing cholangitis in general and IBD populations: a systematic review and meta-analysis
Source: Hepatol Int. 2026 Jul 11;20(5):1463–78. doi: 10.1007/s12072-026-11127-y (PMC13676655; doi:10.1007/s12072-026-11127-y)
Supplement: Supplementary file 6 [file 12072_2026_11127_MOESM4_ESM.docx]

**Supplementary References**

1. Adam H, Alqassas M, Saadah OI, Mosli M. Extraintestinal Manifestations of Inflammatory Bowel Disease in Middle Eastern Patients. *J Epidemiol Glob Health*. 2020;10(4):298-303. doi:10.2991/jegh.k.200330.001
2. Aitola P, Mattila J, Matikainen M. Liver involvement in patients operated for ulcerative colitis, with special reference to the association of cholangitis with colorectal dysplasia and carcinoma. *Int J Colorectal Dis*. 2000;15(3):167-171. doi:10.1007/s003840000223
3. Algaba A, Guerra I, Ricart E, et al. Extraintestinal Manifestations in Patients with Inflammatory Bowel Disease: Study Based on the ENEIDA Registry. *Dig Dis Sci*. 2021;66(6):2014-2023. doi:10.1007/s10620-020-06424-x
4. Bakhshi Z, Hilscher MB, Gores GJ, et al. An update on primary sclerosing cholangitis epidemiology, outcomes and quantification of alkaline phosphatase variability in a population-based cohort. *J Gastroenterol*. 2020;55(5):523-532. doi:10.1007/s00535-020-01663-1
5. Barner-Rasmussen N, Pukkala E, Jussila A, Färkkilä M. Epidemiology, risk of malignancy and patient survival in primary sclerosing cholangitis: a population-based study in Finland. *Scand J Gastroenterol*. 2020;55(1):74-81. doi:10.1080/00365521.2019.1707277
6. Boberg KM, Aadland E, Jahnsen J, Raknerud N, Stiris M, Bell H. Incidence and prevalence of primary biliary cirrhosis, primary sclerosing cholangitis, and autoimmune hepatitis in a Norwegian population. *Scand J Gastroenterol*. 1998;33(1):99-103. doi:10.1080/00365529850166284
7. Boonstra K, Weersma RK, van Erpecum KJ, et al. Population-based epidemiology, malignancy risk, and outcome of primary sclerosing cholangitis. *Hepatology*. 2013;58(6):2045-2055. doi:10.1002/hep.26565
8. Broomé U, Glaumann H, Hellers G, Nilsson B, Sörstad J, Hultcrantz R. Liver disease in ulcerative colitis: an epidemiological and follow up study in the county of Stockholm. *Gut*. 1994;35(1):84-89. doi:10.1136/gut.35.1.84
9. Bruining DH, Siddiki HA, Fletcher JG, Tremaine WJ, Sandborn WJ, Loftus EV Jr. Prevalence of penetrating disease and extraintestinal manifestations of Crohn's disease detected with CT enterography. *Inflamm Bowel Dis*. 2008;14(12):1701-1706. doi:10.1002/ibd.20529
10. Dyson JK, Blain A, Foster Shirley MD, Hudson M, Rushton S, Jeffreys Jones DE. Geo-epidemiology and environmental co-variate mapping of primary biliary cholangitis and primary sclerosing cholangitis. *JHEP Rep*. 2020;3(1):100202. Published 2020 Nov 4. doi:10.1016/j.jhepr.2020.100202
11. Escorsell A, Parés A, Rodés J, Solís-Herruzo JA, Miras M, de la Morena E. Epidemiology of primary sclerosing cholangitis in Spain. Spanish Association for the Study of the Liver. *J Hepatol*. 1994;21(5):787-791. doi:10.1016/s0168-8278(94)80240-8
12. Fedor I, Zold E, Barta Z. Temporal Relationship of Extraintestinal Manifestations in Inflammatory Bowel Disease. *J Clin Med*. 2021;10(24):5984. Published 2021 Dec 20. doi:10.3390/jcm10245984
13. Fernández MIC, Hernández DR, Cabrera Eugenio DE, Palanca W, Guridi ZD, González Fabián L. Diagnosis and Treatment of Autoimmune Liver Diseases in a Tertiary Referral Center in Cuba. *Curr Ther Res Clin Exp*. 2017;85:8-14. Published 2017 Apr 7. doi:10.1016/j.curtheres.2017.04.002
14. Fousekis FS, Katsanos KH, Theopistos VI, et al. Hepatobiliary and pancreatic manifestations in inflammatory bowel diseases: a referral center study. *BMC Gastroenterol*. 2019;19(1):48. Published 2019 Apr 3. doi:10.1186/s12876-019-0967-3
15. Fraga M, Fournier N, Safroneeva E, et al. Primary sclerosing cholangitis in the Swiss Inflammatory Bowel Disease Cohort Study: prevalence, risk factors, and long-term follow-up. *Eur J Gastroenterol Hepatol*. 2017;29(1):91-97. doi:10.1097/MEG.0000000000000747
16. Halling ML, Kjeldsen J, Knudsen T, Nielsen J, Hansen LK. Patients with inflammatory bowel disease have increased risk of autoimmune and inflammatory diseases. *World J Gastroenterol*. 2017;23(33):6137-6146. doi:10.3748/wjg.v23.i33.6137
17. Hashimoto E, Ideta M, Taniai M, et al. Prevalence of primary sclerosing cholangitis and other liver diseases in Japanese patients with chronic ulcerative colitis. *J Gastroenterol Hepatol*. 1993;8(2):146-149. doi:10.1111/j.1440-1746.1993.tb01506.x
18. Heikius B, Niemelä S, Lehtola J, Karttunen T, Lähde S. Hepatobiliary and coexisting pancreatic duct abnormalities in patients with inflammatory bowel disease. *Scand J Gastroenterol*. 1997;32(2):153-161. doi:10.3109/00365529709000186
19. Holzmann K, Klump B, Borchard F, Gregor M, Porschen R. Flow cytometric and histologic evaluation in a large cohort of patients with ulcerative colitis: correlation with clinical characteristics and impact on surveillance. *Dis Colon Rectum*. 2001;44(10):1446-1455. doi:10.1007/BF02234596
20. Juliao-Baños F, Arrubla M, Osorio L, et al. Characterization and prevalence of extraintestinal manifestations in a cohort of patients with inflammatory intestinal disease in Medellin, Colombia. Caracterización y prevalencia de manifestaciones extraintestinales en una cohorte de pacientes con enfermedad inflamatoria intestinal en Medellín (Colombia). *Gastroenterol Hepatol*. 2021;44(6):398-404. doi:10.1016/j.gastrohep.2020.07.012
21. Isene R, Bernklev T, Høie O, et al. Extraintestinal manifestations in Crohn's disease and ulcerative colitis: results from a prospective, population-based European inception cohort. *Scand J Gastroenterol*. 2015;50(3):300-305. doi:10.3109/00365521.2014.991752
22. Kaplan GG, Laupland KB, Butzner D, Urbanski SJ, Lee SS. The burden of large and small duct primary sclerosing cholangitis in adults and children: a population-based analysis. *Am J Gastroenterol*. 2007;102(5):1042-1049. doi:10.1111/j.1572-0241.2007.01103.x
23. Karmiris K, Avgerinos A, Tavernaraki A, et al. Prevalence and Characteristics of Extra-intestinal Manifestations in a Large Cohort of Greek Patients with Inflammatory Bowel Disease. *J Crohns Colitis*. 2016;10(4):429-436. doi:10.1093/ecco-jcc/jjv232
24. Khorashad Khosravi A, Khajedaluee M, Mokhtari Amirmajdi E, et al. Frequency and risk factors of primary sclerosing cholangitis among patients with inflammatory bowel disease in North-East of Iran. *Gastroenterol Hepatol Bed Bench*. 2015;8(3):200-206.
25. Kingham JG, Kochar N, Gravenor MB. Incidence, clinical patterns, and outcomes of primary sclerosing cholangitis in South Wales, United Kingdom. *Gastroenterology*. 2004;126(7):1929-1930. doi:10.1053/j.gastro.2004.04.052
26. Koutroubakis IE, Regueiro M, Schoen RE, et al. Multiyear Patterns of Serum Inflammatory Biomarkers and Risk of Colorectal Neoplasia in Patients with Ulcerative Colitis. *Inflamm Bowel Dis*. 2016;22(1):100-105. doi:10.1097/MIB.0000000000000587
27. Kumagai J, Taida T, Ogasawara S, et al. Clinical characteristics and outcomes of primary sclerosing cholangitis and ulcerative colitis in Japanese patients. *PLoS One*. 2018;13(12):e0209352. Published 2018 Dec 20. doi:10.1371/journal.pone.0209352
28. Lakatos L, Pandur T, David G, et al. Association of extraintestinal manifestations of inflammatory bowel disease in a province of western Hungary with disease phenotype: results of a 25-year follow-up study. *World J Gastroenterol*. 2003;9(10):2300-2307. doi:10.3748/wjg.v9.i10.2300
29. Lamba M, Ngu JH, Stedman CAM. Trends in Incidence of Autoimmune Liver Diseases and Increasing Incidence of Autoimmune Hepatitis. *Clin Gastroenterol Hepatol*. 2021;19(3):573-579.e1. doi:10.1016/j.cgh.2020.05.061
30. Lepistö A, Kärkkäinen P, Järvinen HJ. Prevalence of primary sclerosing cholangitis in ulcerative colitis patients undergoing proctocolectomy and ileal pouch-anal anastomosis. *Inflamm Bowel Dis*. 2008;14(6):775-779. doi:10.1002/ibd.20384
31. Leung KK, Li W, Hansen B, et al. Primary sclerosing cholangitis-inflammatory bowel disease: Epidemiology, mortality, and impact of diagnostic sequence. *JHEP Rep*. 2024;7(3):101272. Published 2024 Nov 30. doi:10.1016/j.jhepr.2024.101272
32. Liang H, Manne S, Shick J, Lissoos T, Dolin P. Incidence, prevalence, and natural history of primary sclerosing cholangitis in the United Kingdom. *Medicine (Baltimore)*. 2017;96(24):e7116. doi:10.1097/MD.0000000000007116
33. Lindkvist B, Benito de Valle M, Gullberg B, Björnsson E. Incidence and prevalence of primary sclerosing cholangitis in a defined adult population in Sweden. *Hepatology*. 2010;52(2):571-577. doi:10.1002/hep.23678
34. Lunder AK, Hov JR, Borthne A, et al. Prevalence of Sclerosing Cholangitis Detected by Magnetic Resonance Cholangiography in Patients With Long-term Inflammatory Bowel Disease. *Gastroenterology*. 2016;151(4):660-669.e4. doi:10.1053/j.gastro.2016.06.021
35. Lupinetti M, Mehigan D, Cameron JL. Hepatobiliary complications of ulcerative colitis. *Am J Surg*. 1980;139(1):113-118. doi:10.1016/0002-9610(80)90239-1
36. Mendes FD, Levy C, Enders FB, Loftus EV Jr, Angulo P, Lindor KD. Abnormal hepatic biochemistries in patients with inflammatory bowel disease. *Am J Gastroenterol*. 2007;102(2):344-350. doi:10.1111/j.1572-0241.2006.00947.x
37. Ngu JH, Gearry RB, Wright AJ, Stedman CA. Inflammatory bowel disease is associated with poor outcomes of patients with primary sclerosing cholangitis. *Clin Gastroenterol Hepatol*. 2011;9(12):1092-e135. doi:10.1016/j.cgh.2011.08.027
38. Nielsen KR, Midjord J, Johannesen HL, Grønbæk H. A nationwide study of autoimmune liver diseases in the Faroe Islands: Incidence, prevalence, and causes of death 2004 - 2021. *Int J Circumpolar Health*. 2023;82(1):2221368. doi:10.1080/22423982.2023.2221368
39. O'Toole A, Alakkari A, Keegan D, Doherty G, Mulcahy H, O'Donoghue D. Primary sclerosing cholangitis and disease distribution in inflammatory bowel disease. *Clin Gastroenterol Hepatol*. 2012;10(4):439-441. doi:10.1016/j.cgh.2011.11.010
40. Olsson R, Danielsson A, Järnerot G, et al. Prevalence of primary sclerosing cholangitis in patients with ulcerative colitis. *Gastroenterology*. 1991;100(5 Pt 1):1319-1323.
41. Ott C, Takses A, Obermeier F, Schnoy E, Müller M. Smoking increases the risk of extraintestinal manifestations in Crohn's disease. *World J Gastroenterol*. 2014;20(34):12269-12276. doi:10.3748/wjg.v20.i34.12269
42. Ozkahraman A, Kayar Y, Dertli R, et al. Extra intestinal manifestations may increase the risk of synchronous and metachronous development of other extraintestinal manifestations in patients with Crohn's disease. *Sci Rep*. 2025;15(1):20629. Published 2025 Jul 1. doi:10.1038/s41598-025-07180-6
43. Park SK, Wong Z, Park SH, et al. Extraintestinal manifestation of inflammatory bowel disease in Asian patients: A multinational study. *Dig Liver Dis*. 2021;53(2):196-201. doi:10.1016/j.dld.2020.06.046
44. Parlak E, Kosar Y, Ulker A, Dagli U, Alkim C, Sahin B. Primary sclerosing cholangitis in patients with inflammatory bowel disease in Turkey. *J Clin Gastroenterol*. 2001;33(4):299-301. doi:10.1097/00004836-200110000-00008
45. Rasmussen HH, Fallingborg J, Mortensen PB, et al. Primary sclerosing cholangitis in patients with ulcerative colitis. *Scand J Gastroenterol*. 1992;27(9):732-736. doi:10.3109/00365529209011174
46. Rasmussen HH, Fallingborg JF, Mortensen PB, Vyberg M, Tage-Jensen U, Rasmussen SN. Hepatobiliary dysfunction and primary sclerosing cholangitis in patients with Crohn's disease. *Scand J Gastroenterol*. 1997;32(6):604-610. doi:10.3109/00365529709025107
47. Rawal KK, Shukla VP, Chikani S, Thakkar M, Ruparelia M, Chudasama RK. Prevalence of extraintestinal manifestations in ulcerative colitis and associated risk factors. *Indian J Gastroenterol*. 2021;40(5):477-482. doi:10.1007/s12664-021-01181-9
48. Roberts H, Rai SN, Pan J, et al. Extraintestinal manifestations of inflammatory bowel disease and the influence of smoking. *Digestion*. 2014;90(2):122-129. doi:10.1159/000363228
49. Rönnblom A, Holmström T, Tanghöj H, Rorsman F, Sjöberg D. Appearance of hepatobiliary diseases in a population-based cohort with inflammatory bowel diseases (Inflammatory Bowel Disease Cohort of the Uppsala Region). *J Gastroenterol Hepatol*. 2015;30(8):1288-1292. doi:10.1111/jgh.12947
50. Schrumpf E, Fausa O, Kolmannskog F, Elgjo K, Ritland S, Gjone E. Sclerosing cholangitis in ulcerative colitis. A follow-up study. *Scand J Gastroenterol*. 1982;17(1):33-39. doi:10.3109/00365528209181041
51. Shepherd HA, Selby WS, Chapman RW, et al. Ulcerative colitis and persistent liver dysfunction. *Q J Med*. 1983;52(208):503-513.
52. Singeap AM, Girleanu I, Diculescu M, et al. Risk Factors for Extraintestinal Manifestations in Inflammatory Bowel Diseases - Data from the Romanian National Registry. *J Gastrointestin Liver Dis*. 2021;30(3):346-357. Published 2021 Sep 21. doi:10.15403/jgld-3818
53. Singh A, Midha V, Narang V, et al. Low prevalence of primary sclerosing cholangitis in patients with inflammatory bowel disease in India. *Intest Res*. 2023;21(4):452-459. doi:10.5217/ir.2022.00087
54. Singh B, Kedia S, Konijeti G, et al. Extraintestinal manifestations of inflammatory bowel disease and intestinal tuberculosis: Frequency and relation with disease phenotype. *Indian J Gastroenterol*. 2015;34(1):43-50. doi:10.1007/s12664-015-0538-7
55. Tan N, Ngu N, Worland T, et al. Epidemiology and outcomes of primary sclerosing cholangitis: an Australian multicentre retrospective cohort study. *Hepatol Int*. 2022;16(5):1094-1104. doi:10.1007/s12072-022-10356-1
56. Tanaka A, Mori M, Matsumoto K, Ohira H, Tazuma S, Takikawa H. Increase trend in the prevalence and male-to-female ratio of primary biliary cholangitis, autoimmune hepatitis, and primary sclerosing cholangitis in Japan. *Hepatol Res*. 2019;49(8):881-889. doi:10.1111/hepr.13342
57. Terg R, Sambuelli A, Coronel E, et al. Prevalence of primary sclerosing cholangitis in patients with ulcerative colitis and the risk of developing malignancies. A large prospective study. *Acta Gastroenterol Latinoam*. 2008;38(1):26-33.
58. Tobias R, Wright JP, Kottler RE, et al. Primary sclerosing cholangitis associated with inflammatory bowel disease in Cape Town, 1975 - 1981. *S Afr Med J*. 1983;63(7):229-235.
59. Toy E, Balasubramanian S, Selmi C, Li CS, Bowlus CL. The prevalence, incidence and natural history of primary sclerosing cholangitis in an ethnically diverse population. *BMC Gastroenterol*. 2011;11:83. Published 2011 Jul 18. doi:10.1186/1471-230X-11-83
60. Trivedi PJ, Crothers H, Mytton J, et al. Effects of Primary Sclerosing Cholangitis on Risks of Cancer and Death in People With Inflammatory Bowel Disease, Based on Sex, Race, and Age. *Gastroenterology*. 2020;159(3):915-928. doi:10.1053/j.gastro.2020.05.049
61. Tse CS, Deepak P, De La Fuente J, et al. Phenotype and Clinical Course of Inflammatory Bowel Disease With Co-existent Celiac Disease. *J Crohns Colitis*. 2018;12(8):973-980. doi:10.1093/ecco-jcc/jjy061
62. Vahedi H, Merat S, Momtahen S, et al. Epidemiologic characteristics of 500 patients with inflammatory bowel disease in Iran studied from 2004 through 2007. *Arch Iran Med*. 2009;12(5):454-460.
63. Vernero M, Saibeni S, Scalvini D, et al. Prevalence and Clinical Impact of Immune-Mediated Inflammatory Diseases in Patients with Inflammatory Bowel Disease: Results from a Large Retrospective Observational Study. *J Clin Med*. 2024;13(4):1019. Published 2024 Feb 9. doi:10.3390/jcm13041019
64. Webb GJ, Ryan RP, Marshall TP, Hirschfield GM. The Epidemiology of UK Autoimmune Liver Disease Varies With Geographic Latitude. *Clin Gastroenterol Hepatol*. 2021;19(12):2587-2596. doi:10.1016/j.cgh.2021.01.029
65. Wei SC, Shieh MJ, Chang MC, Chang YT, Wang CY, Wong JM. Long-term follow-up of ulcerative colitis in Taiwan. *J Chin Med Assoc*. 2012;75(4):151-155. doi:10.1016/j.jcma.2012.02.017
66. Wewer V, Gluud C, Schlichting P, Burcharth F, Binder V. Prevalence of hepatobiliary dysfunction in a regional group of patients with chronic inflammatory bowel disease. *Scand J Gastroenterol*. 1991;26(1):97-102. doi:10.3109/00365529108996489
67. Xu W, Ou W, Guo Y, et al. Clinical outcomes and risk factors of secondary extraintestinal manifestation in ulcerative colitis: results of a multicenter and long-term follow-up retrospective study. *PeerJ*. 2019;7:e7194. Published 2019 Jun 21. doi:10.7717/peerj.7194
68. Yamamoto-Furusho JK, Sánchez-Osorio M, Uribe M. Prevalence and factors associated with the presence of abnormal function liver tests in patients with ulcerative colitis. *Ann Hepatol*. 2010;9(4):397-401.
69. Yang BR, Choi NK, Kim MS, et al. Prevalence of extraintestinal manifestations in Korean inflammatory bowel disease patients. *PLoS One*. 2018;13(7):e0200363. Published 2018 Jul 10. doi:10.1371/journal.pone.0200363
70. Yazdanbod A, Farzaneh E, Pourfarzi F, et al. Epidemiologic profile and clinical characteristics of ulcerative colitis in northwest of Iran: a 10-year review. *Trop Gastroenterol*. 2010;31(4):308-311.
71. Ye BD, Yang SK, Boo SJ, et al. Clinical characteristics of ulcerative colitis associated with primary sclerosing cholangitis in Korea. *Inflamm Bowel Dis*. 2011;17(9):1901-1906. doi:10.1002/ibd.21569
72. Yehuda S, Axlerod R, Toker O, et al. The Association of Inflammatory Bowel Diseases with Autoimmune Disorders: A Report from the epi-IIRN. *J Crohns Colitis*. 2019;13(3):324-329. doi:10.1093/ecco-jcc/jjy166
73. Zeng Z, Zhu Z, Yang Y, et al. Incidence and clinical characteristics of inflammatory bowel disease in a developed region of Guangdong Province, China: a prospective population-based study. *J Gastroenterol Hepatol*. 2013;28(7):1148-1153. doi:10.1111/jgh.12164
74. Zippi M, Corrado C, Pica R, et al. Extraintestinal manifestations in a large series of Italian inflammatory bowel disease patients. *World J Gastroenterol*. 2014;20(46):17463-17467. doi:10.3748/wjg.v20.i46.17463
