# Supplementary material for: Epidemiology of primary sclerosing cholangitis in general and IBD populations: a systematic review and meta-analysis
Source: Hepatol Int. 2026 Jul 11;20(5):1463–78. doi: 10.1007/s12072-026-11127-y (PMC13676655; doi:10.1007/s12072-026-11127-y)
Supplement: Supplementary file 7 [file 12072_2026_11127_MOESM7_ESM.docx]

**Supplementary Table 1.** Summary of cohort characteristics for general and inflammatory bowel disease populations.

| **Author (et al)** | **Year of Publicatio**n | **Study Design** | **Country** | **WHO Region** | **Duration of Study** | **Study Population** |
| --- | --- | --- | --- | --- | --- | --- |
| Adam^1^ | 2020 | Cohort study | Saudi Arabia | EMR | 2013 - 2018 | Patients with confirmed IBD |
| Aitola^2^ | 2000 | Case control study | Finland | EUR | 1982 - 1997 | Patients who underwent proctocolectomy due to ulcerative colitis |
| Algaba^3^ | 2021 | Analytical cross-sectional study | Spain | EUR | 2006 - 2018 | Patients from the Spanish ENEIDA Registry |
| Bakhshi^4^ | 2020 | Cohort study | United States | AMR | 1976 - 2017 | Olmsted country residents |
| Barner-Rasmussen^5^ | 2020 | Cohort study | Finland | EUR | 1990 - 2015 | Hospital District of Helsinki and Uusimaa registry |
| Boberg^6^ | 1998 | Prevalence study | Norway | EUR | 1986 - 1995 | Aker University Hospital in Oslo |
| Boonstra^7^ | 2013 | Cohort study | Netherlands | EUR | 2008 - 2011 | Patients from hospitals in a geographically defined area comprising 50% of Dutch Population |
| Broomé ^8^ | 1994 | Cohort study | Sweden | EUR | 1955 - 1979 | UC patients residing in Stockholm country |
| Bruining^9^ | 2008 | Analytical cross-sectional study | United States | AMR | 2004 - 2005 | CD Patients who underwent computed tomography enterography |
| Dyson^10^ | 2021 | Analytical cross-sectional study | United Kingdom | EUR | N/A | All adult patients with PSC in a tightly defined geographical area within the UK |
| Escorsell^11^ | 1994 | Analytical cross-sectional study | Spain | EUR | 1984 - 1988 | Patients from twenty-three centers from twelve Spanish regions |
| Fedor^12^ | 2021 | Analytical cross-sectional study | Hungary | EUR | 2008 - 2021 | Patients with IBD from a single outpatient clinic |
| Fernandez^13^ | 2017 | Cohort study | Cuba | AMR | 2012 - 2016 | Adult patients admitted to an outpatient clinic |
| Fousekis^14^ | 2019 | Cohort study | Greece | EUR | 1977 - 2016 | Patients with IBD seen at a tertiary university hospital |
| Fraga^15^ | 2017 | Cohort study | Switzerland | EUR | 2006 - 2014 | IBD patients from all regions of Switzerland included in the nationwide SIBDCS |
| Halling^16^ | 2017 | Analytical cross-sectional study | Denmark | EUR | 1977 - 2013 | Patients in the Danish National Patient Registry |
| Hashimoto^17^ | 1993 | Prevalence study | Japan | WPR | 1984 - 1990 | Chronic ulcerative colitis patients diagnosed at Tokyo Women’s Medical College |
| Heikius^18^ | 1997 | Analytical cross-sectional study | Finland | EUR | 1981 - 1991 | Patients with IBD seen at the University Hospital of Oulu |
| Holzmann^19^ | 2001 | Cohort study | Germany | EUR | N/A | Patients with ulcerative colitis |
| Juliao-Baños ^20^ | 2021 | Analytical cross-sectional study | Colombia | AMR | Until 2019 | Adult patients with IBD at the Pablo Tobon Uribe Hospital in Medellin, Colombia |
| Isene^21^ | 2015 | Cohort study | Norway, Netherlands, Denmark, Greece | EUR | 1991 - 2004 | Patients with IBD |
| Kaplan^22^ | 2007 | Cohort study | Canada | AMR | 2000 - 2005 | All residents of the Calgary Health Region |
| Karmiris^23^ | 2016 | Analytical cross-sectional study | Greece | EUR | N/A | Patients with IBD who were followed in eight tertiary Greek hospitals |
| Khorashad^24^ | 2015 | Analytical cross-sectional study | Iran | EMR | 2011 - 2015 | IBD Patients from IBD Clinics of Ghaem and Emam Reza Hospitals |
| Kingham^25^ | 2004 | Cohort study | South Wales | EUR | 1984 - 2003 | All residents of Swansea as demarcated by postal codes |
| Koutroubakis^26^ | 2016 | Cohort study | United States | AMR | 2009 - 2013 | Registry at the IBD Center at the University of Pittsburgh |
| Kumagai^27^ | 2018 | Cohort study | Japan | WPR | 1991 - 2017 | Ulcerative colitis patients diagnosed at Chiba University Hospital |
| Lakatos^28^ | 2003 | Prevalence study | Hungary | EUR | N/A | IBD Patients following up at the Gastroenterology Unit of Csolnoky F. Province Hospital in Veszprem Province |
| Lamba^29^ | 2021 | Cohort study | New Zealand | WPR | 2008 - 2016 | Patients from public hospitals and private practices in Canterbury |
| Lepisto^30^ | 2008 | Cohort study | Finland | EUR | 1993 - 2004 | Patients who underwent proctocolectomy with ileal pouch-anal anastomosis |
| Leung^31^ | 2024 | Cohort study | Canada | AMR | 2002 - 2018 | Health administrative data-derived IBD cohorts from Ontario, Canada |
| Liang^32^ | 2017 | Cohort study | United Kingdom | EUR | 1998 - 2014 | Patients registered with a general practice in the UK Clinical Practice Research Datalink |
| Lindkvist^33^ | 2010 | Cohort study | Sweden | EUR | 1992 - 2005 | Adult population in Vastra Gotaland, a region in southern Sweden |
| Lunder^34^ | 2016 | Prevalence study | Norway | EUR | 1990 - 1993 | Patients in South-Eastern Norway diagnosed with IBD |
| Lupinetti^35^ | 1980 | Cohort study | United States | AMR | 1966 - 1977 | Patients with chronic ulcerative colitis at The John Hopkins Hospital |
| Mendes^36^ | 2007 | Cohort study | United States | AMR | 2000 | All patients with IBD seen for the first time at Mayo Clinic, Rochester, Minnesota |
| Ngu^37^ | 2011 | Cohort study | New Zealand | WPR | 1908 - 2008 | Patients followed up in Christchurch Hospital |
| Nielsen^38^ | 2023 | Cohort study | Faroe Islands | EUR | 2004 - 2021 | Faroese patient registry |
| O'Toole^39^ | 2012 | Analytical cross-sectional study | Ireland | EUR | 1983 - 2011 | IBD Patients attending a single university hospital |
| Olsson^40^ | 1991 | Prevalence study | Sweden | EUR | 1987 - 1988 | Patients with a diagnosis of ulcerative colitis in five well-defined catchment areas, representing 12.7% of Swedish population |
| Ott^41^ | 2014 | Cohort study | Germany | EUR | 2004 - 2008 | Residents in the rural region of Oberpfalz (Bavaria, Germany) |
| Ozkahraman^42^ | 2025 | Cohort study | Turkey | EUR | 1986 - 2011 | Patients with CD followed up in a Turkish hospital |
| Park^43^ | 2021 | Prevalence study | South Korea, Malaysia Myanmar, Thailand, Vietnam | WPR, SEAR | 2000 - 2016 | Patients with IBD recruited from 10 tertiary centers in Asia |
| Parlak^44^ | 2001 | Prevalence study | Turkey | EUR | 1993 - 2000 | Patients with IBD admitted to an IBD reference clinic in Turkey |
| Rasmussen^45^ | 1992 | Prevalence study | Denmark | EUR | 1976 - 1987 | Patients with diagnosis of UC in computerised record system at the Medical and Surgical Center of Gastroenterology in Aalborg Hospital |
| Rasmussen^46^ | 1997 | Prevalence study | Denmark | EUR | 1976 - 1991 | Patients with diagnosis of CD in computerised record system at the Medical and Surgical Center of Gastroenterology in Aalborg Hospital |
| Rawal^47^ | 2021 | Prevalence study | India | SEAR | 2016 - 2017 | Patients with UC seen at a single Indian hospital |
| Roberts^48^ | 2014 | Analytical cross-sectional study | United States | AMR | 1996 - 2013 | Patients with IBD from a single university colorectal surgery practice in the USA |
| Ronnblom^49^ | 2015 | Cohort study | Sweden | EUR | 2005 - 2009 | Patients diagnosed with UC and CD in the Uppsala region in Sweden |
| Schrumpf^50^ | 1982 | Case series study | Norway | EUR | 1974 - 1978 | Patients with UC hospitalized in Medical Dept. A, Rikshospitalet, Oslo |
| Shepherd^51^ | 1983 | Prevalence study | England | EUR | N/A | Patients with UC followed up in an outpatient clinic in Oxford |
| Singeap^52^ | 2021 | Analytical cross-sectional study | Romania | EUR | N/A | Patients with IBD registered in Romanian IBD Prospect National Registry |
| Singh^53^ | 2023 | Prevalence study | India | SEAR | 1991 - 2020 | IBD patients at 5 tertiary care IBD centers in India |
| Singh^54^ | 2015 | Analytical cross-sectional study | India | SEAR | 2005 - 2012 | IBD and intestinal tuberculosis patients seen at a single center study in India |
| Tan^55^ | 2022 | Cohort study | Australia | WPR | 2000 - 2021 | Multicentre study of PSC patients at nine tertiary liver centers across three Australian states |
| Tanaka^56^ | 2019 | Prevalence study | Japan | WPR | 2007 and 2016 | Nationwide hospital-based, epidemiological survey in Japan to identify patients with PBC, PSC and AIH |
| Terg^57^ | 2008 | Cohort study | Argentina | AMR | 1990 - 2003 | Patients with UC seen at a hospital in Hospital de Gastroenterología Dr Bonorino Udaondo in Buenos Aires |
| Tobias^58^ | 1983 | Case series study | South Africa | AFR | N/A | Patients with IBD with raised ALP seen at a hospital in Cape Town |
| Toy^59^ | 2011 | Cohort study | United States | AMR | 2000 - 2006 | Patients with PSC identified from Northern California Kaiser Permanente (KP) database |
| Trivedi^60^ | 2020 | Cohort study | England | EUR | 2006 - 2019 | Patients with IBD, with PSC and without identified from national healthcare registries for adults in England |
| Tse^61^ | 2018 | Case control study | United States | AMR | 1997 - 2016 | Patients with IBD and, co existent IBD and celiac disease in a tertiary referral institution in North America |
| Vahedi^62^ | 2009 | Case series study | Iran | EMR | 2004 - 2007 | Patients with UC and CD referred to tertiary institutions in Tehran |
| Vernero^63^ | 2024 | Analytical cross-sectional study | Italy | EUR | N/A | Patients with IBD in outpatient clinics in two Italian Hospitals |
| Webb^64^ | 2021 | Cohort study | United Kingdom | EUR | 2002 - 2016 | Patients with autoimmune liver conditions identified from UK primary care records |
| Wei^65^ | 2012 | Cohort study | Taiwan | WPR | 1988 - 2008 | Patients with UC and CD identified from chart records in a tertiary medical center in Taiwan |
| Wewer^66^ | 1991 | Analytical cross-sectional study | Denmark | EUR | N/A | Patients with UC and CD identified from outpatient clinic in a Danish hospital |
| Xu^67^ | 2019 | Cohort study | China | WPR | 1986 - 2018 | Patients with UC from prospectively maintained database from Chinese institutes |
| Yamamoto^68^ | 2010 | Cohort study | Mexico | AMR | 2007 - 2008 | Patients with UC identified in a single hospital in Mexico |
| Yang^69^ | 2018 | Prevalence study | South Korea | WPR | 2014 | IBD patients identified from National Health Insurance System Korea |
| Yazdanbod^70^ | 2010 | Analytical cross-sectional study | Iran | EMR | 1998 - 2008 | Patients of a private gastroenterology clinic in Iran with diagnosis of UC at the time of presentation or those whose diagnosis had been made later |
| Ye^71^ | 2011 | Case control study | South Korea | WPR | 1977 - 2009 | Patients diagnosed with UC at tertiary university hospital in Seoul |
| Yehuda^72^ | 2019 | Case control study | Israel | EMR | N/A | IBD patients from 4 healthcare maintenance organizations in Israel |
| Zeng^73^ | 2013 | Cohort study | China | WPR | N/A | Newly diagnosed IBD patients in Zhongshan region China |
| Zippi^74^ | 2014 | Cohort study | Italy | EUR | 2011 - 2012 | Patients with IBD tertiary center in Italy |

**Abbreviations:** WHO: World Health Organisation; AMR: Region of the Americas; EUR: European Region; WPR: Western Pacific Region; SEAR: South-East Asian Region; EMR: Eastern Mediterranean Region; ICD, International Classification of Disease; UC, ulcerative colitis; IBD, inflammatory bowel disease

**Supplementary Table 2.** Quality assessment of included articles using the Joanna Briggs Institute (JBI) Critical Appraisal Tools

**Cohort Studies**

| **Author (et al)** | **Were the two groups similar and recruited from the same population?** | **Were the exposures measured similarly to assign people to both exposed and unexposed groups?** | **Was the exposure measured in a valid and reliable way?** | **Were confounding factors identified?** | **Were strategies to deal with confounding factors stated?** | **Were the groups/participants free of the outcome at the start of the study (or at the moment of exposure)?** | **Were the outcomes measured in a valid and reliable way?** | **Was the follow up time reported and sufficient to be long enough for outcomes to occur?** | **Was follow up complete, and if not, were the reasons to loss to follow up described and explored?** | **Were strategies to address incomplete follow up utilized?** | **Was appropriate statistical analysis used?** |
| --- | --- | --- | --- | --- | --- | --- | --- | --- | --- | --- | --- |
| Adam^1^ | Yes | Yes | Yes | Yes | Yes | No | Yes | Yes | No | No | Yes |
| Bakhshi^4^ | Yes | Yes | Yes | Yes | Yes | Yes | Yes | Yes | N/A | Yes | Yes |
| Barner-Rasmussen^5^ | Yes | Yes | Yes | Yes | Yes | Yes | Yes | Yes | Yes | Yes | Yes |
| Boonstra^7^ | Yes | Yes | Yes | Yes | Yes | Yes | Yes | Yes | Yes | Yes | Yes |
| Broomé ^8^ | Yes | Yes | Yes | Yes | Yes | Yes | Yes | Yes | Yes | Yes | Yes |
| Fernandez^13^ | N/A | Unclear | Yes | Yes | Yes | No | Yes | Yes | Unclear | Yes | Yes |
| Fousekis^14^ | N/A | N/A | Yes | Yes | No | Unclear | Yes | Yes | Unclear | No | Yes |
| Fraga^15^ | Yes | Yes | Yes | Yes | Yes | Yes | Yes | Yes | Unclear | Unclear | Yes |
| Holzmann^19^ | Yes | Yes | Yes | Yes | No | Unclear | Yes | Yes | Unclear | No | Yes |
| Isene^21^ | Yes | Yes | Yes | Yes | No | Yes | Yes | Yes | Yes | No | Yes |
| Kaplan^22^ | Yes | Yes | Yes | Yes | Yes | Yes | Yes | N/A | N/A | N/A | Yes |
| Kingham^25^ | Yes | Yes | Yes | Yes | No | Yes | Yes | Yes | Yes | Yes | Yes |
| Koutroubakis^26^ | Yes | Yes | Yes | Yes | Yes | Yes | Yes | Yes | No | No | Yes |
| Kumagai^27^ | Yes | Yes | Yes | Yes | Yes | Yes | Yes | Yes | No | Yes | Yes |
| Lamba^29^ | Yes | Yes | Yes | Yes | No | Yes | Yes | Yes | Yes | Yes | Yes |
| Lepisto^30^ | Yes | Yes | Yes | Yes | No | Unclear | Yes | Yes | Unclear | N/A | Yes |
| Leung^31^ | Yes | Yes | Yes | Yes | Yes | Yes | Yes | Yes | Yes | Yes | Yes |
| Liang^32^ | Yes | Yes | Yes | Yes | Yes | Yes | Yes | Yes | Yes | Yes | Yes |
| Lindkvist^33^ | N/A | N/A | N/A | Yes | Yes | Yes | Yes | Yes | Yes | Yes | Yes |
| Lupinetti^35^ | Yes | Yes | Yes | Yes | No | Unclear | Yes | Yes | Unclear | No | Yes |
| Mendes^36^ | Yes | Yes | Yes | Yes | Yes | Unclear | Yes | Yes | No | No | Yes |
| Ngu^37^ | Yes | Yes | Yes | Yes | No | Yes | Yes | Yes | Unclear | N/A | Yes |
| Nielsen^38^ | N/A | N/A | N/A | No | No | Yes | Yes | Yes | Unclear | Unclear | Yes |
| Ott^41^ | Yes | Yes | No | Yes | Yes | No | Yes | Yes | Unclear | N/A | Yes |
| Ozkahraman^42^ | N/A | N/A | N/A | Yes | Yes | No | Yes | Yes | No | No | Yes |
| Ronnblom^49^ | Yes | Yes | Yes | Unclear | No | No | Yes | Yes | No | No | Yes |
| Tan^55^ | N/A | N/A | Yes | Yes | Yes | Unclear | Yes | Yes | Unclear | N/A | Yes |
| Terg^57^ | Yes | Yes | Yes | Yes | Yes | Yes | Yes | Yes | Unclear | N/A | Yes |
| Toy^59^ | Yes | Yes | Yes | Yes | Yes | Yes | Yes | Yes | Unclear | N/A | Yes |
| Trivedi^60^ | Yes | Yes | Yes | Yes | Yes | Yes | Yes | Yes | Yes | Yes | Yes |
| Webb^64^ | Yes | Yes | Yes | Yes | Yes | Yes | Yes | Yes | Yes | Yes | Yes |
| Wei^65^ | Yes | N/A | N/A | N/A | No | Yes | Yes | Yes | Unclear | N/A | Yes |
| Xu^67^ | Yes | Yes | Yes | Yes | Yes | Yes | Yes | Yes | Yes | No | Yes |
| Yamamoto^68^ | Yes | Yes | Yes | Yes | Yes | Yes | Yes | Yes | Unclear | N/A | Yes |
| Zeng^73^ | Yes | N/A | N/A | N/A | N/A | Yes | Yes | Yes | No | Yes | Yes |
| Zippi^74^ | Yes | Yes | Yes | Unclear | No | Yes | Yes | Yes | Unclear | N/A | Yes |

**Analytical Cross-Sectional Studies**

| **Author (et al)** | **Were the criteria for inclusion in the sample clearly defined?** | **Were the study subjects and the setting described in detail?** | **Was the exposure measured in a valid and reliable way?** | **Were objective, standard criteria used for measurement of the condition?** | **Were confounding factors identified?** | **Were strategies to deal with confounding factors stated?** | **Were the outcomes measured in a valid and reliable way?** | **Was appropriate statistical analysis used?** |
| --- | --- | --- | --- | --- | --- | --- | --- | --- |
| Algaba^3^ | Yes | Yes | Yes | Yes | Yes | Yes | Yes | Yes |
| Bruining^9^ | Yes | Yes | Yes | Yes | Yes | No | Yes | Yes |
| Dyson^10^ | Yes | Yes | Yes | Yes | Yes | Yes | Yes | Yes |
| Escorsell^11^ | Yes | Yes | Yes | Yes | Yes | No | Yes | Yes |
| Fedor^12^ | Yes | Yes | Yes | Yes | Yes | Yes | Yes | Yes |
| Halling^16^ | Yes | Yes | Yes | Yes | Yes | Yes | Yes | Yes |
| Heikius^18^ | Yes | Yes | Yes | Yes | Yes | No | Yes | Yes |
| Juliao-Baños ^20^ | Yes | Yes | Yes | Yes | Yes | No | Yes | Yes |
| Karmiris^23^ | Yes | Yes | Yes | Yes | Yes | Yes | Yes | Yes |
| Khorashad^24^ | Yes | Yes | Yes | Yes | Yes | No | Yes | Yes |
| O'Toole^39^ | Yes | Yes | Yes | Yes | Yes | Yes | Yes | Yes |
| Roberts^48^ | Yes | Yes | Yes | Yes | Yes | Yes | Unclear | Yes |
| Singeap^52^ | Yes | Yes | Yes | Yes | Yes | Yes | Unclear | Yes |
| Singh^53^ | Yes | Yes | Yes | Yes | Unclear | No | Yes | Yes |
| Vernero^63^ | Yes | Yes | Yes | Yes | Yes | Yes | Yes | Yes |
| Wewer^66^ | Yes | Yes | Yes | Yes | Yes | No | Yes | Yes |
| Yazdanbod^70^ | Yes | Yes | Yes | Yes | Yes | No | Yes | Yes |

**Prevalence Studies**

| **Author (et al)** | **Was the sample frame appropriate to address the target population?** | **Were study participants sampled in an appropriate way?** | **Was the sample size adequate?** | **Were the study subjects and the setting described in detail?** | **Was the data analysis conducted with sufficient coverage of the identified sample?** | **Were valid methods used for the identification of the condition?** | **Was the condition measured in a standard, reliable way for all participants?** | **Was there appropriate statistical analysis?** | **Was the response rate adequate, and if not, was the low response rate managed appropriately?** |
| --- | --- | --- | --- | --- | --- | --- | --- | --- | --- |
| Boberg^6^ | Yes | Yes | Yes | Yes | Yes | Yes | Yes | Yes | N/A |
| Hashimoto^17^ | Yes | Yes | Yes | Yes | Yes | Yes | Yes | Yes | N/A |
| Lakatos^28^ | Yes | Yes | Yes | Yes | Yes | Yes | Yes | Yes | Yes |
| Lunder^34^ | Yes | Yes | Yes | Yes | Yes | Yes | Yes | Yes | Yes |
| Olsson^40^ | Yes | Yes | Yes | Yes | Yes | Yes | Yes | Yes | Yes |
| Park^43^ | Unclear | Unclear | Yes | Yes | Yes | Yes | Yes | Yes | N/A |
| Parlak^44^ | Unclear | Yes | Yes | Yes | Yes | Yes | No | No | Unclear |
| Rasmussen^45^ | Yes | Yes | Yes | Yes | No | Yes | No | Yes | No |
| Rasmussen^46^ | Yes | Yes | Yes | Yes | Yes | Yes | Yes | Yes | Yes |
| Rawal^47^ | Unclear | Yes | Yes | Yes | Yes | Yes | Yes | Yes | Unclear |
| Shepherd^51^ | Yes | Yes | Yes | Yes | Yes | Yes | Yes | Yes | N/A |
| Singh^54^ | Yes | Yes | Yes | Yes | Yes | Yes | Yes | Yes | N/A |
| Tanaka^56^ | Yes | Yes | Yes | Yes | Yes | Yes | Yes | Yes | Yes |
| Yang^69^ | Yes | Yes | Yes | Yes | Yes | Yes | Yes | Yes | N/A |

**Case Control Studies**

| **Author (et al)** | **Were the groups comparable other than the presence of disease in cases or the absence of disease in controls?** | **Were cases and controls matched appropriately?** | **Were the same criteria used for identification of cases and controls?** | **Was exposure measured in a standard, valid and reliable way?** | **Was exposure measured in the same way for cases and controls?** | **Were confounding factors identified?** | **Were strategies to deal with confounding factors stated?** | **Were outcomes assessed in a standard, valid and reliable way for cases and controls?** | **Was the exposure period of interest long enough to be meaningful?** | **Was appropriate statistical analysis used?** |
| --- | --- | --- | --- | --- | --- | --- | --- | --- | --- | --- |
| Aitola^2^ | Yes | Yes | Yes | Yes | Yes | Yes | Yes | Yes | Yes | Yes |
| Tse^61^ | Yes | Yes | Yes | Yes | Yes | Yes | Yes | Yes | Yes | Yes |
| Ye^71^ | Yes | Yes | Yes | Yes | Yes | Yes | Yes | Yes | Yes | Yes |
| Yehuda^72^ | Yes | Yes | Yes | Yes | Yes | Yes | Yes | Yes | Yes | Yes |

**Case Series Studies**

| **Author (et al)** | **Were there clear criteria for inclusion in the case series?** | **Was the condition measured in a standard, reliable way for all participants included in the case series?** | **Were valid methods used for identification of the condition for all participants included in the case series?** | **Did the case series have consecutive inclusion of participants?** | **Did the case series have complete inclusion of participants?** | **Was there clear reporting of the demographics of the participants in the study?** | **Was there clear reporting of clinical information of the participants?** | **Were the outcomes or follow up results of cases clearly reported?** | **Was there clear reporting of the presenting site(s)/clinic(s) demographic information?** | **Was statistical analysis appropriate?** |
| --- | --- | --- | --- | --- | --- | --- | --- | --- | --- | --- |
| Schrumpf^50^ | Yes | Yes | Yes | Yes | Yes | Yes | Yes | Yes | Yes | Yes |
| Tobias^58^ | Yes | Yes | Yes | Unclear | Unclear | Yes | Yes | Yes | Yes | N/A |
| Vahedi^62^ | Yes | Yes | Yes | Yes | Yes | Yes | Yes | Unclear | Yes | Yes |

**Supplementary Table 3.** Prevalence of Primary Sclerosing Cholangitis in Ulcerative Colitis Population

|  | **No. of studies** | **Sample Size** | **Cases** | **Pooled prevalence per 1,000**  **[95% confidence interval]** | **I^2^** | **Prediction interval**^††^ | **Subgroup difference** |
| --- | --- | --- | --- | --- | --- | --- | --- |
| **Overall** | 47 | 101,276 | 1,096 | 20.6 [15.2 – 27.8] | 96.6% [96.0 – 97.1] | 2.7 – 141.9 |  |
|  |  |  |  |  |  |  |  |
| **Sensitivity Analyses** |  |  |  |  |  |  |  |
| Excluding pre-2018 administrative studies | 42 | 66,745 | 809 | 21.0 [15.2 – 28.9] | 96.0% [95.3 – 96.6] | 2.7 – 143.9 |  |
| Excluding ICD only studies | 44 | 34,593 | 738 | 22.7 [16.8 – 30.7] | 92.4% [90.7 – 93.8] | 3.2 – 143.3 |  |
| High-quality studies (JBI ≥ 80%) | 38 | 98,594 | 1,023 | 20.1 [14.3 – 28.4] | 97.1% [96.5 – 97.5] | 2.4 – 148.4 |  |
|  |  |  |  |  |  |  |  |
| **Phenotype** |  |  |  |  |  |  |  |
| Small-Duct PSC | 9 | 5,499 | 27 | 3.0 [0.9 - 10.6] | 85.1% | 0.1 – 145.4 | p = 0.001* |
| Large-Duct PSC | 9 | 5,499 | 145 | 27.0 [17.8 - 40.9] | 75.9% | 6.6 – 104.2 |  |
|  |  |  |  |  |  |  |  |
| **Ulcerative Colitis Subtype** |  |  |  |  |  |  |  |
| Proctitis | 7 | 1,026 | 5 | 4.9 [2.0 – 11.7] | 0.0% | 1.5 – 15.3 | p < 0.001* |
| Pancolitis | 11 | 3,841 | 207 | 55.9 [41.7 – 74.7] | 70.5% | 20.0 – 146.5 |  |
| Left-Sided | 8 | 2,438 | 30 | 6.8 [2.0 – 22.6] | 0.0% | 0.2 – 184.6 |  |
| Others | 4 | 1,022 | 27 | 27.9 [9.6 - 78.8] | 86.4% | -^†^ |  |
|  |  |  |  |  |  |  |  |
| **Gender** |  |  |  |  |  |  |  |
| Male | 10 | 20,797 | 180 | 22.5 [12.1 – 41.6] | 96.8% [95.5 – 97.7] | 2.3 – 189.3 | p < 0.001* |
| Female | 10 | 16,020 | 108 | 14.0 [8.3 – 23.5] | 90.2% [84.2 - 94.0] | 2.4 – 77.2 | p < 0.001* |
|  |  |  |  |  |  |  |  |
| **WHO Region^#^** |  |  |  |  |  |  |  |
| Eastern Mediterranean Region | 4 | 919 | 66 | 66.1 [36.9 – 115.4] | 78.1% | -^†^ | p < 0.001* |
| European Region | 25 | 51,734 | 667 | 21.7 [14.6 – 32.1] | 96.5% | 2.8 – 146.5 |  |
| Region of the Americas | 6 | 3,321 | 131 | 38.3 [27.2 – 53.6] | 71.8% | 13.4 – 104.5 |  |
| South-East Asia Region | 3 | 10,604 | 48 | 4.5 [3.4 – 6.0] | 0.0% | -^†^ |  |
| Western Pacific Region | 7 | 33,318 | 167 | 11.3 [5.8 – 21.9] | 96.1% | 1.3 – 90.7 |  |
| African Region | 1 | 250 | 8 | 32.0 [16.1 – 62.7] | -** | -^†^ |  |
|  |  |  |  |  |  |  |  |
| **Socio-Demographic Index** |  |  |  |  |  |  |  |
| High | 28 | 77,039 | 774 | 21.3 [14.4 – 31.5] | 97.4% | 2.6 – 155.1 | p < 0.001* |
| High-Middle | 10 | 11,207 | 219 | 29.1 [17.0 – 49.4] | 94.6% | 3.9 – 184.7 |  |
| Middle | 6 | 2,426 | 55 | 20.9 [9.6 – 44.9] | 83.8% | 1.6 – 216.0 |  |
| Low-Middle | 3 | 10,604 | 48 | 4.5 [3.4 – 6.0] | 0.0% | -^†^ |  |
|  |  |  |  |  |  |  |  |
| **Dates** |  |  |  |  |  |  |  |
| Before 2018 | 35 | 50,842 | 780 | 25.1 [18.2 - 34.5] | 95.7% | 3.9 – 145.5 | p = 0.036* |
| 2018 Onwards | 12 | 50,434 | 316 | 11.8 [6.3 - 22.1] | 96.8% | 1.0 – 122.0 |  |
|  |  |  |  |  |  |  |  |
| **Case-Ascertainment Method** |  |  |  |  |  |  |  |
| Administrative database studies | 7 | 65,371 | 396 | 12.3 [5.4 – 27.7] | 93.4% | 0.6 – 199.7 | p = 0.165 |
| Hospital/ Clinic-based Studies | 40 | 35,905 | 700 | 22.8 [16.7 – 31.2] | 98.1% | 3.3 – 142.6 |  |
|  |  |  |  |  |  |  |  |
| **Diagnosis Method** |  |  |  |  |  |  |  |
| Imaging | 15 | 16,920 | 293 | 26.2 [15.4 – 44.2] | 95.2% | 2.8 – 202.6 | p < 0.001* |
| Imaging and Biopsy | 18 | 11,334 | 331 | 26.7 [17.4 – 40.8] | 90.3% | 3.9 – 159.6 |  |
| ICD Code | 3 | 66,683 | 358 | 6.0 [3.4 – 10.6] | 96.8% | -^†^ |  |
| Others/ Unspecified | 11 | 6,339 | 114 | 14.0 [7.7 – 25.3] | 88.7% | 1.7 – 104.6 |  |

**Legend:** *: p<0.05 denotes statistical significance; **: I^2^ not calculated for single-study subgroups; ^†^: Prediction intervals not reported for subgroups with k<5 studies due to unreliable estimation of between-study variance; ^††^: Prediction intervals should be interpreted with caution due to substantial between-study heterogeneity, which may result in imprecise estimation of between-study variance and wide prediction bounds. **^#^** One study excluded for recruiting patients from centers across both Western Pacific and South-east Asian WHO regions.

**Abbreviations:** WHO, World Health Organisation; JBI, Joanna Briggs Institute

**Supplementary Table 4.** Prevalence of Primary Sclerosing Cholangitis in Crohn’s Disease Population

|  | **No. of studies** | **Sample Size** | **Cases** | **Pooled prevalence per 1,000**  **[95% confidence interval]** | **I^2^** | **Prediction interval**^††^ | **Subgroup difference** |
| --- | --- | --- | --- | --- | --- | --- | --- |
| **Overall** | 31 | 46,725 | 252 | 10.2 [6.6 – 15.8] | 91.1% [88.4 – 93.1] | 1.0 – 93.4 |  |
|  |  |  |  |  |  |  |  |
| **Sensitivity Analyses** |  |  |  |  |  |  |  |
| Excluding pre-2018 administrative studies | 27 | 31,362 | 191 | 10.0 [6.4 – 15.7] | 87.6% [83.2 – 90.9] | 1.2 – 80.2 |  |
| Excluding ICD only studies | 28 | 13,093 | 138 | 11.9 [7.6 – 18.8] | 82.5% [75.7 – 87.5] | 1.3 – 99.9 |  |
| High-quality studies (JBI ≥ 80%) | 25 | 45,471 | 229 | 9.4 [5.7 – 15.7] | 92.2% [89.7 – 94.1] | 0.8 – 101.6 |  |
|  |  |  |  |  |  |  |  |
| **Phenotype** |  |  |  |  |  |  |  |
| Small-Duct PSC | 4 | 818 | 14 | 15.5 [6.0 - 39.4] | 0.0% | -^†^ | p = 0.671 |
| Large-Duct PSC | 4 | 818 | 16 | 21.3 [6.8 - 64.5] | 85.3% | -^†^ |  |
|  |  |  |  |  |  |  |  |
| **Crohn’s Disease Subtype** |  |  |  |  |  |  |  |
| Ileal | 3 | 508 | 5 | 9.8 [4.1 – 23.4] | 46.4% | -^†^ | p = 0.378 |
| Ileocolonic | 3 | 520 | 13 | 34.0 [9.3 – 116.9] | 87.9% | -^†^ |  |
| Colonic | 4 | 1,227 | 9 | 6.2 [0.8 – 47.1] | 85.4% | -^†^ |  |
| Other | 3 | 866 | 13 | 15.0 [8.7 – 25.7] | 0.0% | -^†^ |  |
|  |  |  |  |  |  |  |  |
| **Gender** |  |  |  |  |  |  |  |
| Male | 6 | 10,912 | 37 | 9.7 [3.3 – 28.5] | 93.2% [88.0 – 96.2] | 0.3 – 264.0 | p < 0.001* |
| Female | 6 | 5,404 | 27 | 7.4 [2.6 – 20.7] | 85.3% [70.0 – 92.8] | 0.3 – 150.9 | p < 0.001* |
|  |  |  |  |  |  |  |  |
| **WHO Region^#^** |  |  |  |  |  |  |  |
| Eastern Mediterranean Region | 3 | 397 | 7 | 15.9 [4.6 – 53.5] | 0.0% | -^†^ | p < 0.001* |
| European Region | 19 | 27,301 | 186 | 13.7 [8.0 – 23.3] | 92.5% | 1.3 – 131.1 |  |
| Region of the Americas | 3 | 1,045 | 16 | 15.3 [9.4 – 24.8] | 0.0% | -^†^ |  |
| South-East Asia Region | 2 | 3,242 | 6 | 1.9 [0.8 – 4.1] | 0.0% | -^†^ |  |
| Western Pacific Region | 2 | 13,942 | 33 | 2.4 [1.7 – 3.3] | 0.0% | -^†^ |  |
| African Region | 1 | 164 | 2 | 12.2 [3.1 – 47.4] | -** | -^†^ |  |
|  |  |  |  |  |  |  |  |
| **Socio-Demographic Index** |  |  |  |  |  |  |  |
| High | 16 | 33,605 | 157 | 11.8 [6.1 – 22.9] | 94.8% | 0.7 – 165.9 | p < 0.001* |
| High-Middle | 9 | 8,863 | 82 | 13.3 [8.3 – 21.1] | 73.8% | 3.5 – 49.4 |  |
| Middle | 4 | 1,015 | 7 | 7.1 [3.0 – 16.7] | 6.0% | -^†^ |  |
| Low-Middle | 2 | 3,242 | 6 | 1.9 [0.8 – 4.1] | 0.0% | -^†^ |  |
|  |  |  |  |  |  |  |  |
| **Dates** |  |  |  |  |  |  |  |
| Before 2018 | 21 | 20,542 | 139 | 12.7 [7.0 – 22.7] | 91.3% | 1.0 – 144.7 | p = 0.078 |
| 2018 Onwards | 10 | 26,183 | 113 | 6.3 [3.8 – 10.5] | 83.1% | 1.2 – 32.2 |  |
|  |  |  |  |  |  |  |  |
| **Case-Ascertainment Method** |  |  |  |  |  |  |  |
| Administrative database studies | 6 | 30,387 | 102 | 8.4 [2.9 – 24.2] | 96.5% | 0.2 – 298.9 | p = 0.636 |
| Hospital/ Clinic-based Studies | 25 | 16,338 | 150 | 11.1 [7.0 – 17.6] | 81.1% | 1.4 – 84.2 |  |
|  |  |  |  |  |  |  |  |
| **Diagnosis Method** |  |  |  |  |  |  |  |
| Imaging | 10 | 6,077 | 56 | 13.8 [6.3 – 30.1] | 89.1% | 0.8 – 190.5 | p = 0.008* |
| Imaging and Biopsy | 10 | 4,026 | 52 | 12.5 [5.2 – 29.6] | 85.5% | 0.6 – 203.9 |  |
| ICD Code | 3 | 33,632 | 114 | 3.5 [2.0 – 6.3] | 93.6% | -^†^ |  |
| Others/ Unspecified | 8 | 2,990 | 30 | 10.0 [7.0 – 14.4] | 0.0% | 6.2 – 16.2 |  |

**Legend:** *: p<0.05 denotes statistical significance; **: I^2^ not calculated for single-study subgroups; ^†^: Prediction intervals not reported for subgroups with k<5 studies due to unreliable estimation of between-study variance; ^††^: Prediction intervals should be interpreted with caution due to substantial between-study heterogeneity, which may result in imprecise estimation of between-study variance and wide prediction bounds. **^#^** One study excluded for recruiting patients from centers across both Western Pacific and South-east Asian WHO regions.

**Abbreviations:** WHO, World Health Organisation; JBI, Joanna Briggs Institute

**Supplementary Table 5.** Incidence of Primary Sclerosing Cholangitis in General Population

|  | **No. of studies** | **Events** | **Person-Years** | **Pooled Incidence Rate per 100,000 Person-Years**  **[95% confidence interval]** | **I^2^** | **Prediction interval**^††^ | **Subgroup Analysis** |
| --- | --- | --- | --- | --- | --- | --- | --- |
| **Overall** | 13 | 2,206 | 274,365,833 | 0.892 [0.705 – 1.130] | 97.2% [96.3 – 97.9] | 0.353 – 2.254 |  |
|  |  |  |  |  |  |  |  |
| **Sensitivity Analyses** |  |  |  |  |  |  |  |
| Excluding pre-2018 administrative studies | 11 | 1,704 | 188,828,485 | 0.995 [0.797 – 1.243] | 96.8% [95.6 – 97.7] | 0.445 – 2.227 |  |
| High-quality studies (JBI ≥ 80%) | 12 | 2,199 | 273,365,833 | 0.904 [0.706 – 1.158] | 97.4% [96.6 – 98.1] | 0.345 – 2.369 |  |
|  |  |  |  |  |  |  |  |
| **WHO Region** |  |  |  |  |  |  |  |
| European Region | 8 | 1,968 | 239,812,725 | 0.889 [0.662 – 1.190] | 98.1% | 0.314 – 2.510 | p = 0.683 |
| Region of the Americas | 3 | 183 | 28,950,242 | 0.792 [0.412 – 1.525] | 95.5% | -^†^ |  |
| Western Pacific Region | 2 | 55 | 5,602,866 | 1.113 [0.659 – 1.880] | 54.2% | -^†^ |  |
|  |  |  |  |  |  |  |  |
| **Socio-Demographic Index** |  |  |  |  |  |  |  |
| High | 13 | 2,206 | 274,365,833 | 0.892 [0.705 – 1.130] | 97.2% [96.3 – 97.9] | 0.353 – 2.254 |  |
|  |  |  |  |  |  |  |  |
| **Dates** |  |  |  |  |  |  |  |
| Before 2018 | 8 | 1,120 | 173,207,137 | 0.839 [0.601 - 1.172] | 95.2% | 0.256 – 2.749 | p = 0.429 |
| 2018 Onwards | 5 | 1,086 | 101,158,696 | 1.008 [0.740 – 1.372] | 97.1% | 0.327 – 3.105 |  |
|  |  |  |  |  |  |  |  |
| **Case-Ascertainment Method** |  |  |  |  |  |  |  |
| Administrative database studies | 4 | 905 | 139,837,348 | 0.599 [0.448 – 0.802] | 87.7% | -^†^ | p = 0.004* |
| Hospital/ Clinic-based Studies | 9 | 1,301 | 134,528,485 | 1.057 [0.824 – 1.356] | 97.0% | 0.440 – 2.539 |  |
|  |  |  |  |  |  |  |  |
| **Diagnosis Method** |  |  |  |  |  |  |  |
| Imaging | 4 | 803 | 55,311,475 | 1.314 [1.051 - 1.643] | 78.6% | -^†^ | p < 0.001* |
| Imaging and Biopsy | 7 | 586 | 99,973,108 | 0.823 [0.583 – 1.163] | 92.9% | 0.245 – 2.765 |  |
| Others/ Unspecified | 2 | 817 | 119,081,250 | 0.689 [0.596 - 0.798] | 78.0% | -^†^ |  |

**Legend:** *: p<0.05 denotes statistical significance; ^†^: Prediction intervals not reported for subgroups with k<5 studies due to unreliable estimation of between-study variance; ^††^: Prediction intervals should be interpreted with caution due to substantial between-study heterogeneity, which may result in imprecise estimation of between-study variance and wide prediction bounds.

**Abbreviations:** WHO, World Health Organisation; JBI, Joanna Briggs Institute

**Supplementary Table 6.** Meta Regression Evaluating Factors associated with Prevalence of Primary Sclerosing Cholangitis in Ulcerative Colitis Patients

| **Variable** | **Odds Ratio** | **95% Confidence Interval** | **P-Value** |
| --- | --- | --- | --- |
| **WHO Region [Reference: South-East Asia Region]** |  |  |  |
| European Region | 5.335 | 1.637 – 17.385 | p = 0.005* |
| Eastern Mediterranean Region | 15.701 | 3.713 – 66.393 | p < 0.001* |
| Region of the Americas | 8.605 | 2.256 – 32.819 | p = 0.002* |
| African Region | 7.389 | 0.885 – 61.676 | p = 0.065 |
| Western Pacific Region | 2.843 | 0.748 – 10.804 | p = 0.125 |
|  |  |  |  |
| **Socio-Demographic Index [Reference: High-Middle]** |  |  |  |
| High | 0.752 | 0.364 – 1.550 | p = 0.439 |
| Middle | 0.772 | 0.266 – 2.238 | p = 0.634 |
| Low-Middle | 0.144 | 0.036 – 0.572 | p = 0.005* |
|  |  |  |  |
| **Case-Ascertainment Method [Reference: Administrative database studies]** |  |  |  |
| Hospital/ Clinic-based Studies | 1.949 | 0.863 – 4.401 | p = 0.108 |
|  |  |  |  |
| **Diagnosis Method [Reference: Imaging and Biopsy]** |  |  |  |
| Imaging | 1.009 | 0.515 – 1.976 | p = 0.977 |
| ICD Code | 0.211 | 0.068 – 0.651 | p = 0.007* |
| Others/ Unspecified | 0.547 | 0.256 – 1.167 | p = 0.119 |
|  |  |  |  |
| **Phenotype [Reference: Large-Duct PSC]** |  |  |  |
| Small-Duct PSC | 0.192 | 0.063 – 0.588 | p = 0.004* |
|  |  |  |  |
| **Dates [Reference: 2018 Onwards]** |  |  |  |
| Before 2018 | 2.141 | 1.091 – 4.205 | p = 0.027* |

**Legend:** *: p<0.05 denotes statistical significance

**Supplementary Table 7.** Meta Regression Evaluating Factors associated with Prevalence of Primary Sclerosing Cholangitis in Crohn’s Disease Patients

| **Variable** | **Odds Ratio** | **95% Confidence Interval** | **P-Value** |
| --- | --- | --- | --- |
| **WHO Region [Reference: South-East Asian Region]** |  |  |  |
| European Region | 8.299 | 1.289 – 53.412 | p = 0.026* |
| Eastern Mediterranean Region | 8.061 | 0.774 – 84.011 | p = 0.081 |
| Region of the Americas | 8.100 | 0.913 – 71.842 | p = 0.060 |
| African Region | 6.411 | 0.320 – 128.484 | p = 0.224 |
| Western Pacific Region | 2.306 | 0.192 – 27.632 | p = 0.510 |
|  |  |  |  |
| **Socio-Demographic Index [Reference: High-Middle]** |  |  |  |
| High | 0.939 | 0.363 – 2.425 | p = 0.896 |
| Middle | 0.673 | 0.150 – 3.014 | p = 0.605 |
| Low-Middle | 0.127 | 0.017 – 0.931 | p = 0.042* |
|  |  |  |  |
| **Case-Ascertainment Method [Reference: Administrative database studies]** |  |  |  |
| Hospital/ Clinic-based Studies | 1.596 | 0.587 – 4.338 | p = 0.359 |
|  |  |  |  |
| **Diagnosis Method [Reference: Imaging and Biopsy]** |  |  |  |
| Imaging | 0.996 | 0.372 – 2.669 | p = 0.994 |
| ICD Code | 0.212 | 0.057 – 0.791 | p = 0.021* |
| Others/ Unspecified | 0.681 | 0.231 – 2.004 | p = 0.485 |
|  |  |  |  |
| **Phenotype [Reference: Large-Duct PSC]** |  |  |  |
| Small-Duct PSC | 0.827 | 0.166 – 4.121 | p = 0.816 |
|  |  |  |  |
| **Dates [Reference: 2018 Onwards]** |  |  |  |
| Before 2018 | 2.412 | 1.045 – 5.569 | p = 0.039* |

**Legend:** *: p<0.05 denotes statistical significance
